# A full Bayesian partition model for identifying hypo- and hyper-methylated loci from single nucleotide resolution sequencing data

**DOI:** 10.1186/s12859-015-0850-3

**Published:** 2016-01-11

**Authors:** Henan Wang, Chong He, Garima Kushwaha, Dong Xu, Jing Qiu

**Affiliations:** Department of Statistics, University of Missouri, Columbia, Missouri USA; Department of Computer Science and Informatics Institute, Columbia, USA; Department of Applied Economics and Statistics, University of Delaware, Newark, DE USA

**Keywords:** DNA methylation, Full Bayesian partition model, Hypo-methylation, Hyper-methylation

## Abstract

**Backgroud:**

DNA methylation is an epigenetic modification that plays important roles on gene regulation. Study of whole-genome bisulfite sequencing and reduced representation bisulfite sequencing brings the availability of DNA methylation at single CpG resolution. The main interest of study on DNA methylation data is to test the methylation difference under two conditions of biological samples. However, the high cost and complexity of this sequencing experiment limits the number of biological replicates, which brings challenges to the development of statistical methods.

**Results:**

Bayesian modeling is well known to be able to borrow strength across the genome, and hence is a powerful tool for high-dimensional- low-sample- size data. In order to provide accurate identification of methylation loci, especially for low coverage data, we propose a full Bayesian partition model to detect differentially methylated loci under two conditions of scientific study. Since hypo-methylation and hyper-methylation have distinct biological implication, it is desirable to differentiate these two types of differential methylation. The advantage of our Bayesian model is that it can produce one-step output of each locus being either equal-, hypo- or hyper-methylated locus without further post-hoc analysis. An R package named as MethyBayes implementing the proposed full Bayesian partition model will be submitted to the bioconductor website upon publication of the manuscript.

**Conclusions:**

The proposed full Bayesian partition model outperforms existing methods in terms of power while maintaining a low false discovery rate based on simulation studies and real data analysis including bioinformatics analysis.

**Electronic supplementary material:**

The online version of this article (doi:10.1186/s12859-015-0850-3) contains supplementary material, which is available to authorized users.

## Background

DNA methylation is methylation of cytosine residues at CpG dinucleotides in a DNA sequence and affects 70–80 % of all CpG dinucleotides in mammals [1]. It is the most widely studied epigenetic modification and is known to have profound effects on gene expression. It is involved in embryogensis, genomic imprinting [2], X-chromosome inactivation [3], and many diseases [4], particularly various types of cancers [5]. DNA methylation change in cancer tissue compared to normal tissue can be both increased, called hyper-methylation, or decreased, called hypo-methylation. DNA hyper-methylation is shown to be present at more specific locations, like CpG Islands, compared to the more diffused hypo-methylation all over the genome. CpG island and their methylation are not only present in promoters and upstream regions of genes, but also within gene bodies known as gene-body methylation.

Given the influence of methylation on the gene expression, there are a lot of studies aiming to identify differentially methylated loci in diseased tissue samples compared to their respective normal samples. Among the methods developed to quantify the (relative) levels of CpG methylation in the whole genome, bisulfite sequencing is a common technique and has its advantages. This method involves treating DNA with sodium bisulfite [6], which converts un-methylated cytosines to uracil and leaves methylated cytosines unchanged. Treated DNA is then used to generate high-throughtput readouts by DNA sequencing technique. It can provide methylation level at a single nucleotide resolution.

The availability of this bisulfite sequencing (BS-seq), together with the influence of the DNA methylation on human disease, has led to extensive studies in detecting differentially methylated loci (DML) based on the case and control study. Several statistical methods have been applied to test DML. Fisher exact test [7] is a commonly used statistical approach for testing DML by pooling sequencing reads among the individuals in each condition. BSmooth [8] considers the variation among biological replicates, and uses a signal-to-noise statistics similar to the *t* statistics to discern differential methylation region via a smoothing approach across genome for each individual under two conditions. When considering the binary feature of each locus being methylated or not, Methylkit [9] utilizes a logistic regression model where a condition effect is incorporated to identify DML between the normal and cancer condition. Since logistic regression can be sensitive to small sample size, a filtering of the data is recommended before analysis so that only loci with at least 10 reads coverage for each sample are included for the analysis which will guarantee the overall sample size, the summation of read coverage over all samples in each condition, is large enough for the logistic regression to have good power. The DSS package [10] proposes an empirical Bayes Wald test to identify DML for single nucleotide resolution sequencing data. They consider a beta-binomial model to take into account of the biological variation that might exist for the methylation proportions and use an empirical Bayes approach to estimate the associated dispersion parameter. However the null distribution of the derived test statistics is unknown, although a normal distribution is recommended in their paper purely based on simulation studies.

All the above statistical approaches face a common problem that they only produce binary output of a locus being differentially methylated or not. However, for DNA methylation data, it is important to differentiate hypo- and hyper-methylation because they have very different biological implication. For instance, DNA hyper-methylation are usually associated with transcriptional inactivation of cancer-related genes by increased methylation in CpG island (regions with high CpG density) in their promoter region [11–13]. In contrast, DNA hypo-methylation is shown to be present within repeated DNA elements [14] and is linked to chromosomal instability, loss of imprinting and oncogene activation (eg. c-Myc). A common practice to further identify hypo- or hyper-methylated loci is based on the sign of the test statistics, which is an ad-hoc approach and ignores the uncertainty associated with the test statistics. In this article, we propose a full Bayesian partition model to identify hypo- and hyper-methylated loci simultaneously without further post-hoc analysis. In the proposed method, we introduce a latent variable representing the methylation group membership at each locus and the statistical inference is based on the posterior distribution of this latent variable with the final outcome of the analysis being whether a locus is an equal-, hypo- or hyper-methylated locus. In addition to producing one-step outputs, the Bayesian approach is also well-known to be able to borrow strength across the genome, and hence can be more powerful for small sample size study, which is fairly common for the DNA methylation studies due to the high cost of BS-seq experiments [10].

The rest of this article is organized as follows. In the Methods section, we introduce the proposed Bayesian model and implement a Metropolis-Hastings algorithm to obtain the posterior samples of genome-wide group membership for posterior inference. The section of simulation studies and the section of real data analysis present simulation studies and real data analysis including bioinformatics analysis respectively to evaluate the performance of the proposed Bayesian approach. The advantage of the proposed approach is shown by comparing with several competing methods. The discussion of the proposed method and future work is presented in the discussion section.

## Methods

### Model

We propose a full Bayesian partition model to identify DML based on a case-control study. Suppose there are a total of *L* CpG loci, with a number of *n*_1_ samples in the case study and *n*_2_ samples in the control study. Let *C*_*ijk*_ and *M*_*ijk*_ denote the read coverage and the number of methylated reads respectively at the *i*th locus (*i*=1,2,…,*L*) of the *j*th (*j*=1,2,…,*n*_*k*_) replicate of the *k*th condition (*k*=1 for case, *k*=2 for control). Let *p*_*ik*_ be the true methylation proportion for the *i*th locus of the *k*th condition. For each locus *i*, since there are two kinds of reads, methylated and non-methylated, we model the distribution of the number of methylated reads by the following binomial distribution: *M*_*ijk*_|*C*_*ijk*_,*p*_*ik*_∼Binomial(*C*_*ijk*_,*p*_*ik*_).

In this study, since we are interested in identifying both hypo- and hyper-methylated loci, the *L* CpG loci are partitioned into three groups: group 0 containing equal-methylated loci where *p*_*i*1_=*p*_*i*2_; group 1 containing hypo-methylated loci where *p*_*i*1_<*p*_*i*2_ and group 2 containing hyper-methylated loci where *p*_*i*1_>*p*_*i*2_. We introduce a *L*-dimensional latent indicator variable ***I***=(*I*_1_,…,*I*_*L*_) with *I*_*i*_=0,1,2 to indicate the three possible group memberships of different loci. Let *l*_0_,*l*_1_ and *l*_2_ denote the number of loci in each of the three groups (*l*_0_+*l*_1_+*l*_2_=*L*). Let the data matrix of read coverages for group 0 be $\boldsymbol {C}_{0}=\left [\boldsymbol {c}_{i_{1}}, \boldsymbol {c}_{i_{2}}, \dots, \boldsymbol {c}_{i_{l_{0}}}\right ]$ where $\boldsymbol {c}_{i}=\left [C_{i11}, \dots, C_{{in}_{1}1}, C_{i12}, \dots, C_{{in}_{2}2}\right ]^{\prime }$ representing the vector of read coverages for the *i*th locus, consisting of observations for both case and control conditons, and $i_{1}, \dots, i_{l_{0}}$ are indices for loci in group 0. Similarly, let ***C***_1_,***C***_2_ denote the data matrices of read coverage for group 1 and 2 respectively, and ***M***_0_,***M***_1_,***M***_2_ denote the data matrices of numbers of methylated reads for group 0, 1, and 2 respectively.

Assuming independence among loci, we describe the likelihood model as follows. Let ***Θ***_0_={(*p*_*i*1_,*p*_*i*2_):*I*_*i*_=0} be the methylation proportions for loci in group 0. Then the likelihood of ***M***_0_ can be expressed as

(1) $${} p(\boldsymbol{M}_{0}| \boldsymbol{C}_{0}, \boldsymbol{\Theta}_{0})=\prod_{i: I_{i}=0} \prod_{k=1}^{2}\prod_{j=1}^{n_{k}} {C_{ijk} \choose M_{ijk}} p_{ik}^{M_{ijk}}(1-p_{ik})^{C_{ijk}-M_{ijk}}.   $$

We consider a conjugate beta prior distribution for the true methylation proportions *p*_*ik*_. Note for loci in group 0, *p*_*i*1_=*p*_*i*2_. Hence we consider the joint prior density function for (*p*_*i*1_,*p*_*i*2_) in ***Θ***_0_ as follows:

(2) $$\begin{array}{*{20}l} p\left(p_{i1}, p_{i2}| \alpha_{1}, \beta_{1}, I_{i}=0\right)=f\left(p_{i1}; \alpha_{1}, \beta_{1}\right)1_{(p_{i1}=p_{i2})} \end{array} $$

where 1_(.)_ is an indicator function and $f(p; \alpha, \beta)=\frac {1}{B(\alpha, \beta)} p^{\alpha -1}(1-p)^{\beta -1}$ with $B(\alpha, \beta)=\frac {\Gamma (\alpha)\Gamma (\beta)} {\Gamma (\alpha +\beta)}$ and $\Gamma (\alpha)=\int _{0}^{\infty }x^{\alpha -1}e^{-x}dx$. After integrating out ***Θ***_0_, we obtain the marginal probability

(3) $${} {\fontsize{8.8pt}{9.6pt}{\begin{aligned} p(\boldsymbol{M}_{0}| \boldsymbol{C}_{0}, \boldsymbol{I})&=\prod_{\{ i: I_{i}=0\}}\left[\prod_{k=1}^{2} \prod_{j=1}^{n_{k}} \frac{C_{ijk}!}{M_{ijk}!N_{ijk}!}\right]\\&\quad \frac{B\left(\alpha_{1}+\sum_{k=1}^{2}\sum_{j=1}^{n_{k}}M_{ijk},\beta_{1}+\sum_{k=1}^{2}\sum_{j=1}^{n_{k}}N_{ijk}\right)}{B(\alpha_{1},\beta_{1})}\\ \end{aligned}}}  $$

where *N*_*ijk*_=*C*_*ijk*_−*M*_*ijk*_.

Loci in group 1 are hypo-methylated with *p*_*i*1_<*p*_*i*2_. Therefore, we consider a joint truncated beta distribution by adjusting the prior distribution proposed by [15] for two ordered means in the setting of microarray data analysis. Let ***Θ***_1_={(*p*_*i*1_,*p*_*i*2_):*I*_*i*_=1}. Then the joint prior density function for (*p*_*i*1_,*p*_*i*2_) in ***Θ***_1_ is

(4) $$ \begin{aligned} &p(p_{i1}, p_{i2}|\alpha_{2}, \beta_{2}, I_{i}=1)=2f(p_{i1}; \alpha_{2}, \beta_{2})\\&f(p_{i2}; \alpha_{2}, \beta_{2})1_{(p_{i1}<p_{i2})}.  \end{aligned}  $$

Note that the likelihood function of ***M***_1_ given ***Θ***_1_ and ***C***_1_ is similar to Eq. (1). After integrating out ***Θ***_1_ using the prior density (4), we obtain the marginal probability

(5) $$ {} \begin{aligned} p(\boldsymbol{M}_{1}| \boldsymbol{C}_{1}, \boldsymbol{I})=&\prod_{\{i: I_{i}=1\}}\left\{ \prod_{k=1}^{2}\left[\prod_{j=1}^{n_{k}}\frac{C_{ijk}!}{N_{ijk}!M_{ijk}!}\right]\right. \\&\left. \frac{B\left(\sum_{j=1}^{n_{k}}M_{ijk}+\alpha_{2},\sum_{j=1}^{n_{k}}N_{ijk}+\beta_{2}\right)}{B\left(\alpha_{2},\beta_{2}\right)}\right\} \\ &\times 2P(X_{i}<Y_{i}) \end{aligned}  $$

where $X_{i}\sim Beta\left (\alpha _{2}+\sum _{j=1}^{n_{1}}M_{ij1},\beta _{2}+\sum _{j=1}^{n_{1}}N_{ij1}\right)$ and is independent of $Y_{i}\sim Beta\left (\alpha _{2}+\sum _{j=1}^{n_{2}}M_{ij2},\beta _{2}+\sum _{j=1}^{n_{2}}N_{ij2}\right)$.

For loci in group 2, the direction of the methylation proportion comparison between the case and control conditions is reversed. Let ***Θ***_2_={(*p*_*i*1_,*p*_*i*2_):*I*_*i*_=2}. Then the joint prior density function for (*p*_*i*1_,*p*_*i*2_) in ***Θ***_2_ is

(6) $$ \begin{aligned} &p(p_{i1}, p_{i2}|\alpha_{3}, \beta_{3}, I_{i}=2)=2f(p_{i1}; \alpha_{3}, \beta_{3})\\&f(p_{i2}; \alpha_{3}, \beta_{3})1_{(p_{i1}>p_{i2})}. \end{aligned}  $$

By integrating out ***Θ***_2_, we can obtain the marginal probability

(7) $$ \begin{aligned} p(\boldsymbol{M}_{2}| \boldsymbol{C}_{2}, \boldsymbol{I})=&\prod_{\{i: I_{i}=2\}}\left\{ \prod_{k=1}^{2}\left[\prod_{j=1}^{n_{k}}\frac{C_{ijk}!}{N_{ijk}!M_{ijk}!}\right]\right.\\& \left. \frac{B\left(\sum_{j=1}^{n_{k}}M_{ijk}+\alpha_{3},\sum_{j=1}^{n_{k}}N_{ijk}+\beta_{3}\right)}{B(\alpha_{3},\beta_{3})}\right\} \\ &\times 2P(X_{i}>Y_{i})  \end{aligned}  $$

where $X_{i}\sim Beta\left (\alpha _{3}+\sum _{j=1}^{n_{1}}M_{ij1},\beta _{3}+\sum _{j=1}^{n_{1}}N_{ij1}\right)$ and is independent of $Y_{i}\!\!\sim \!\! Beta\left (\alpha _{3}+\sum _{j=1}^{n_{2}}M_{ij2},\beta _{3}\,+\right.$$\left.\sum _{j=1}^{n_{2}}N_{ij2}\right)$.

To make inference about the membership of each locus along the whole genome, the posterior distribution of ***I*** can be obtained by

(8) $$ p(\boldsymbol{I}|\boldsymbol{C}, \boldsymbol{M}) \propto p(\boldsymbol{M}_{0}|\boldsymbol{C}_{0}, \boldsymbol{I})p(\boldsymbol{M}_{1}|\boldsymbol{C}_{1}, \boldsymbol{I})p(\boldsymbol{M}_{2}| \boldsymbol{C}_{2}, \boldsymbol{I})p(\boldsymbol{I}).  $$

(See Additional file 1 for the derivation). Since *I*_*i*_ for *i*=1,…,*L* has three possible entries, the prior distribution of *I*_*i*_ is modeled by multinomial (1; *π*_0_,*π*_1_,*π*_2_) with ***π***=(*π*_0_,*π*_1_,*π*_2_) to be the vector of probabilities of belonging to the three groups. Then *L*-vector ***I*** has density $p(\boldsymbol {I} |\boldsymbol {\pi })=\pi _{0}^{l_{0}}\pi _{1}^{l_{1}}\pi _{2}^{l_{2}}$, where $l_{j}=\sum _{i=1}^{L} 1_{\{I_{i}=j\}}$ with *j*=0,1,2. We further set a Dirichlet prior for parameters ***π***: $p(\pi _{0}, \pi _{1}|k_{0},k_{1},k_{2})=\frac {1}{B(k_{0}, k_{1}, k_{2})} \prod _{i=0}^{2}\pi _{i}^{(k_{i}-1)}$, where *π*_2_=1−*π*_0_−*π*_1_ and obtain that

(9) $$ \begin{aligned} p(\boldsymbol{I})&=\int p(\boldsymbol{I}|\boldsymbol{\pi})p(\boldsymbol{\pi}|k_{0},k_{1},k_{2})d\boldsymbol{\pi} \\ &=\frac {B\left(k_{0}+l_{0}, k_{1}+l_{1}, k_{2}+l_{2}\right)}{B\left(k_{0}, k_{1}, k_{2}\right)}. \end{aligned}  $$

where $B(k_{0}, k_{1}, k_{2})=\frac {\Gamma (k_{0})\Gamma (k_{1})\Gamma (k_{2})}{\Gamma (k_{0}+k_{1}+k_{2})}$. Thus, the posterior distribution of ***I*** in (8) can be obtained by combining the formulas (3), (5), (7) and (9).

### Model fitting via MCMC

We investigate the posterior distribution of *L* dimensional classification variable ***I*** using Markov chain Monte Carlo (MCMC) techniques [16], and in lack of conjugacy, a Metropolis Hastings sampling algorithm is applied to sample posterior draws of ***I*** in (8). Before we implement MCMC procedures, hyperparameter values in priors need to be specified. Beta priors in three groups are chosen to be non-informative, i.e., *α*_1_,*α*_2_,*α*_3_,*β*_1_,*β*_2_ and *β*_3_ are all set to be ones. Dirichlet prior is also chosen to be non-informative with *k*_0_,*k*_1_ and *k*_2_ being ones. We first randomly assign an initial state for each locus and then iteratively generate samples of ***I*** from its full conditional posterior distribution by the following steps. We define ***I***^*old*^ to be current membership vector at the previous MCMC iteration, and ***I***^*new*^ to be the proposed sample of new membership vector. First, we randomly choose one of the following two proposals for new value of ***I***: *(a)* Randomly pick one methylation group and choose a locus from this group, then change its membership value to one of other memberships; or *(b)* Randomly pick two methylation groups and choose a locus from each of these two groups from the ***I***^*old*^ and exchange their membership values. Second, the proposed value ***I***^*new*^ will be accepted based on the Metropolis Hastings probability:

(10) $$ \text{min} \left\{1, \frac {p\left(\boldsymbol{I}^{new}|\boldsymbol{C}, \boldsymbol{M}\right)}{p\left(\boldsymbol{I}^{old}|\boldsymbol{C}, \boldsymbol{M}\right)}\times \frac {p\left(\boldsymbol{I}^{new}\rightarrow \boldsymbol{I}^{old}\right)}{p\left(\boldsymbol{I}^{old}\rightarrow \boldsymbol{I}^{new}\right)}\right\}  $$

where *p*(***I***|***C***,***M***) is the posterior density of ***I*** in (8), and *p*(***I***^*old*^→***I***^*new*^) is a transition probability from ***I***^*old*^ to ***I***^*new*^, i.e., the probability of generating ***I***^*new*^ from ***I***^*old*^ based on the above two proposals.

Post-burn-in MCMC samples are used to draw posterior inference, and the classification of each locus into equal-, hypo-, or hyper-methylated group can be made by using marginal posterior distribution. We calculate the empirical frequency of each locus belonging to each group by summarizing the Markov chain Monte Carlo output, and divide this empirical frequency by the total post-burn-in sample size to obtain the estimates of posterior membership probabilities for *i*=1, …, L. The methylation status of each locus is chosen as the membership group with highest posterior probability estimates. We tried different initial values of I and our simulation studies show that the posterior inference is robust to the choice of initial values.

## Simulation studies

### Simulation settings

We conducted two sets of simulation studies to evaluate the accuracy of the proposed method in identification of methylated loci, and compare the proposed procedure with some existing ones such as logistic regression model [9], *z*-test comparing two proportions, and empirical Bayes Wald test [10]. Here we didn’t compare with the BSmooth method [8] because it was proposed for detecting differentially methylated region, not for DML. As stated in the user’s guide of their package, the BSmooth algorithm depends heavily on smoothing and requires the methylation levels to be measured in bigger region of the genome, instead of single loci. Hence it was excluded from our comparison in this paper.

In order to best mimic the structure of real methylation data, we generated data based on a real dataset [17] where a genome-wide DNA methylation analysis for 11 CD 19^+^ B-cells from chronic lymphocytic leukemia (CLL) patients and 3 normal control samples were conducted to identify DML. For all case and control samples, methylation data were generated using reduced representation bisulfite sequencing (RRBS) at single-base resolution. For our simulation study, we selected two case and two control samples out of all samples in their real dataset. We selected only two samples for each condition to represent the common situation of small sample size often associated with DNA methylation data due to the high cost of BS-seq experiments. Also, as it is known that the variation can differ significantly among cancer samples [18], to minimize the sample variation among the samples in the case study so that it is justifiable to ignore the subject effect, we selected two CLL samples with matched percent identity, CD38 percent and IGHV mutation status. We arbitrary choose two control samples out of the three. Total 384,890 loci are commonly mapped by these two case samples and two control samples.

We first identified the three groups (equal, hypo- and hyper- methylated groups) of loci based on the real data using different cutoff values and then generate parameters for simulated data from the empirical distributions of the parameter estimates of each identified group. We calculated the maximum likelihood estimates of methylation proportions ($\hat {p}_{\textit {ik}}=\sum _{j} M_{\textit {ijk}} / \sum _{j} C_{\textit {ijk}}$) under each condition (case and control), and computed the difference $\hat {d}_{i}=\hat {p}_{i1}-\hat {p}_{i2}$. Loci with $|\hat {d}_{i}|<0.005$ are classified as equal-methylated group, loci with $\hat {d}_{i}>\gamma $ are classified as hyper-methylated group and loci with $\hat {d}_{i}< -\gamma $ are classified as hypo-methylated group, where the positive value *γ* is a tuning parameter to control the effect sizes for different simulation settings. The empirical distributions of the parameter estimators $\hat {p}_{\textit {ik}}$ for each of the three identified groups were used to generate true parameters *p*_*ik*_ for the simulated data. When *γ* is small, the empirical distribution of $\hat {d}_{i}$ for the hypo- and hyper-methylated groups will cover more smaller values and hence the corresponding simulated data will have more smaller differential methylation levels for the hyper- and hypo- methylated groups. On the other hand, when *γ* is large, the empirical distribution of $\hat {d}_{i}$ for the hypo and hyper methylated groups will cover more larger values and hence the resultant simulated data have more larger signals. Therefore, we expect the power of various procedures is larger for large *γ* and smaller for small *γ*. (Note that *γ* is a tuning parameter to control the effect sizes for different simulation settings. We set different values of *γ* for simulation studies to see how our procedure performs in various situations. There is no such value for real data analysis. The only input for our procedure in real data analysis is the real data. There is no tuning parameter that need to be specified by the users).

The value of *γ* also determines the proportions of the three groups for the simulated data. For instance, when *γ*=0.01, there are 43.28 % hypo-methylated loci and 29.44 % hyper-methylated loci based on the real data. While when *γ*=0.2, there are 5.9 % hypo-methylated loci and 4.6 % hyper-methylated loci. For our simulated data, we generated three groups of loci according to these proportions. We considered different values of *γ*, *γ*=0.01,0.05,0.1,0.15,0.2, to study the effects of the signal strength and percentage of signals in various simulation settings. Additional file 1: Table S1 gives the proportion of hypo- and hyper-methylated loci for the simulated data at different values of *γ*.

In the first set of simulation studies, we assumed *p*_*ik*_ does not depend on the replicates. For equal-methylated loci, we used the pooled estimates of the common methylation proportion of the real data as the true proportion: $\sum _{j,k}M_{\textit {ijk}} / \sum _{j,k}C_{\textit {ijk}}$. For hypo- (hyper-) methylated loci, the methylation proportion of the real data is estimated by $\sum _{j=1}^{n_{1}}{M_{ij1}}/\sum _{j=1}^{n_{1}}{C_{ij1}} $ and $\sum _{j=1}^{n_{2}}{M_{ij2}}/\sum _{j=1}^{n_{2}}{ C_{ij2}} $ separately for each of the case and control conditions, and is then used as the true methylation proportion *p*_*ik*_ for the simulated data.

Our model assumed that the methylation proportion is the same for biological replicates within the case or control condition. However, in reality, biological variation might exist for the methylation proportions [10]. Therefore, we conducted further simulation studies to test the robustness of our model when such biological variation exists. We refer to the second set of simulation studies as *simulation studies with subject effect* (as a contrast, the first simulation setting is called the *simulation studies without subject effect*). To be specific, we calculated the estimated methylation proportion for each patient of the real data $\hat {p}_{\textit {ijk}}=M_{\textit {ijk}}/C_{\textit {ijk}} $ and used it as the true parameter to generate the methylated counts from the following binomial distribution *M*_*ijk*_∼binomial(*C*_*ijk*_,*p*_*ijk*_), where *C*_*ijk*_ is the observed read coverage of the real data for the selected loci. Even though we allow the methylation proportion to vary across replicates within the case or control condition, the CpG loci can be still classified into equal- or hypo- or hyper-methylated groups based on the observed difference in methylation proportions between the case and control conditions as described in the first simulation setting.

Although our Bayesian model assumes beta prior distributions for the methylation proportions, our simulation studies try to mimic the real data as much as possible without making any parametric distribution for the methylation proportions. As described earlier in this section, the parameter *p*_*ik*_ is generated from the empirical distribution of the methylation proportion estimates of the real data. Here we consider the simulation results when there are two replicates per condition since it is quite common for this type of data to have small sample sizes [10]. For each simulation set, we generated a total of 20,000 loci, and the proportion of hypo- (or hyper-) methylated loci in the simulation data is determined by the tuning parameter *γ* (see Additional file 1: Table S1 for the detailed numbers).

### Simulation results

We compared our Bayesian partition model to three existing methods: (1) the logistic regression model of Methylkit package [9]; (2) the standard *z*-test comparing two proportions; and (3) an empirical Bayes Wald test [10]. Note that a standard practice is to apply a two-sample *t*-test to the individual methylation proportion estimates *M*_*ijk*_/*C*_*ijk*_ [10]. However, with two replicates per condition, it works very poorly. Hence we replaced it with the standard *z*-test for comparing two proportions which pooled sequencing reads among individuals under each condition. Note that all the three existing methods produce a *p*-value for each CpG locus and hence need to be adjusted for multiplicity. We applied the *q*-value approach in [19] to control false discovery rate at a nominal level. Since our Bayesian model makes inference based on the posterior probability and doesn’t produce *p*-values, there is no FDR control. In order to make a fair comparison, we evaluated the actual FDR level of the Bayesian inference in the simulation studies and then used it as the nominal level of the FDR control for the other three methods.

Our Bayesian model produces three types of loci for each simulated data while the other three methods only produce binary results: DML or non-DML. Therefore, for these approaches, the direction of differential methylation is decided based on the signs of the estimates of the methylation proportion difference between the case and control conditions. Since for simulated data, it is known whether a locus is equal-, hypo- or hyper-methylated, we can evaluate different rates of false positives and true positives. We summarized our simulation results averaging over 100 simulation runs using five measures: FDR, mdFDR, TPR, TPR_hypo and TPR_hyper. Here the false discovery rate (FDR) is calculated without considering the misclassification between hypo- and hyper-methylated loci and defined as the proportion of truly non-DML among those identified as differentially methylated, while the mixed directional FDR (mdFDR) [20] considers the misspecification between the hypo- and hyper-methylated loci as false discoveries in addition to the mistakes of classifying non-DML as DML. The true positive rate (TPR) is defined as the proportion of truly DML that are detected by the method without considering the misspecification between the hypo- and hyper-methylated loci, while TPR for hypo-methylated loci (TPR_hypo) is defined as the proportion of truly hypo-methylated loci that are detected by the method. Similarly, TPR for hyper-methylated loci (TPR_hyper) is defined as the proportion of truly hyper-methylated loci that are detected by the method.

The results of the simulation studies without subject effect are reported in Table 1. Although our Bayesian inference does not control the FDR, Table 1 shows that the actual FDR level of our approach is very small and stable (ranges from 0.01 to 0.02 for different values of *γ*). Even when directional mistakes (namely hyper-methylated loci are declared as hypo-methylated or vice versa) are taken into account, the mdFDR of the proposed Bayesian method is still reasonably small, ranges from 0.01 to 0.03, and often equals to the FDR (in other words, there are zero directional mistakes, see Additional file 1: Table S2).

**Table 1:** Comparison of performance in terms of FDR, mdFDR, TPR, TPR_hypo and TPR_hyper of four procedures (logistic regression, DSS, *z*-test and the proposed Bayesian) at different *γ* values for the first set of simulation studies (without subject effect). Results are averaged over 100 replications of 20,000 CpG loci with two samples under each condition

		Logistic	DSS	*z*-test	Bayesian
		regression			
*γ*=0.01	FDR	0.0043	0.0001	0.0055	0.0091
	mdFDR	0.0048	0.0001	0.0059	0.0109
	TPR	0.2156	0.0837	0.2027	0.2548
	TPR_hypo	0.2162	0.0752	0.2129	0.2596
	TPR_hyper	0.2145	0.0952	0.1889	0.2471
*γ*=0.05	FDR	0.0158	0.0003	0.0182	0.0184
	mdFDR	0.0159	0.0003	0.0183	0.0188
	TPR	0.3861	0.1672	0.3528	0.4099
	TPR_hypo	0.3921	0.159	0.3694	0.4251
	TPR_hyper	0.3787	0.1772	0.3326	0.3912
*γ*=0.1	FDR	0.0207	0.0005	0.0244	0.0216
	mdFDR	0.0207	0.0005	0.0244	0.0216
	TPR	0.5398	0.2934	0.4964	0.5775
	TPR_hypo	0.5379	0.2851	0.5011	0.5879
	TPR_hyper	0.542	0.3032	0.4909	0.5652
*γ*=0.15	FDR	0.0208	0.0005	0.0251	0.0217
	mdFDR	0.0208	0.0005	0.0251	0.0217
	TPR	0.6691	0.4345	0.6307	0.7161
	TPR_hypo	0.6619	0.422	0.627	0.7201
	TPR_hyper	0.6779	0.4497	0.6353	0.7113
*γ*=0.2	FDR	0.02	0.0006	0.0257	0.0214
	mdFDR	0.02	0.0006	0.0257	0.0214
	TPR	0.7769	0.5751	0.7476	0.8214
	TPR_hypo	0.7708	0.5628	0.7422	0.8253
	TPR_hyper	0.7848	0.591	0.7546	0.8163

When the FDR of other procedures is controlled at the actual FDR level of the Bayesian approach using the *q*-value approach [19], our approach always outperforms other approaches in terms of power for different values of *γ*, whether the power is in terms of TPR, TPR_hypo or TPR_hyper. In terms of power, the ranking of the four approaches is very clear: the Bayesian approach works the best, the second one is the logistic regression approach, the third one is *z*-test and the last one is the empirical Bayes Wald test of the DSS package [10]. The power improvement of the proposed Bayesian approach over the logistic regression model, *z* test and the DSS procedure can be as high as 20 *%*, 30 *%* and 250 *%* respectively for small *γ* and can still be more than 7 *%*, 11 *%* and 46 *%* respectively for large *γ*. (See Additional file 1: Table S3 for the relative power improvement of the proposed approach over other methods for different *γ*). An interesting observation is that the power of the proposed approach to detect hypo-methylated loci (TPR_hypo) is always larger than its power to detect the hyper-methylated loci (TPR_hyper). This is likely due to the fact that there are more hypo-methylated loci than the hyper-methylated loci in the simulated data, which has the same proportion of differential methylation as the real data. (See Additional file 1: Table S1 for the proportions of hypo- and hyper-methylated loci of the real data for different values of *γ*).

As expected, the power of all procedures increases with the value *γ* since the magnitude of signal is larger and hence easier to detect even though the proportion of signal in the data are decreasing. For instance, when *γ*=0.01, the power of the proposed method is about 25 *%*, while when *γ*=0.2, the power of the proposed method is about 82 *%*. This pattern is observed for all other procedures as well. However, a big surprise to us is the poor performance of the empirical Bayes Wald test (also called DSS) since it claims to borrow strength for better dispersion parameter estimates in their beta-binomial setting and should work well. A closer look at the actual FDR of this procedure tells us that this procedure is very conservative in its FDR control. The actual FDRs of the logistic regression model and *z*-test are close to their nominal levels, which is the actual FDR of the proposed Bayesian method. However, the actual FDR of the DSS procedure is significantly below the nominal level. A possible explanation might be that the null distribution of the DSS test statistics is no longer approximately normal in our simulation setting, which can be seen from the normal quantile quantile (QQ) plot of the DSS test statistics in Additional file 1: Figure S1, where all the CpG loci belong to the equal-methylated group in one simulated data and hence the graph represents the null distribution of the DSS test statistics. The QQ plot shows that the two tails of the null distribution of the DSS test not only deviate from the normal distribution but they are not very symmetric. Even if one thinks the normal distribution is a good approximation to the null distribution of the DSS test, one needs to be careful about what values to use for the mean and the standard deviation of this normal distribution. DSS [10] did not address this issue in their paper, but in their package (version 2.5.3.), the p-value of the DSS test was calculated assuming that the null distribution of the test statistics was a standard normal distribution (the authors also confirmed it through personal communication). However, the histogram of the DSS test statistics in Additional file 1: Figure S1 clearly shows that the null distribution of the DSS test deviates from the standard normal with variance smaller than one. Since we calculate the p-value of the DSS test based on the standard normal distribution as implemented in their package, it is not surprising that the null distribution of the *p*-values in Additional file 1: Figure S2 is skewed to the left and the test result is very conservative.

The results of the simulation studies with subject effect are reported in Table 2, Additional file 1: Table S4 and Additional file 1: Table S5. These numbers tell the same story as Table 1, Additional file 1: Table S2 and Additional file 1: Table S3. The actual FDR of the Bayesian approach is still reasonable small and stable for different values of *γ* and it ranges from 0.01 to 0.02 and the mdFDR is almost always equal to FDR, implying zero directional mistakes most of the time. The power of the proposed method to detect hypo-methylated loci is always higher than the one to detect hyper-methylated loci because there are more hypo-methylated loci in the data. The power of the proposed method ranges from around 25 *%* to 82 *%* for various values of *γ*. And for all values of *γ*, the proposed Bayesian approach has the best power among the four procedures under consideration with the power improvement over the other three approaches as high as over 30 *%*, 40 *%* and 1425 *%* respectively. Similar to the case without subject effect, the DSS procedure still performs poorly under the simulation setting with subject effect. The histogram in Additional file 1: Figure S3 shows that the null distribution of the DSS test statistics deviates even more from the standard normal distribution(with variance even smaller than one) than in the case without subject effect. Therefore, the *p*-values calculated assuming the standard normal distribution for the null distribution as implemented in the DSS package (version 2.5.3) tend to be very large (see the histogram of the *p*-values under the null hypothesis in right panels of Additional file 1: Figure S2) and lead to very conservative results. In summary, the results produced for simulation studies with subject effect are similar to those for simulation studies without subject effect. This might indicate that the two case samples we have chosen from the real data with matched covariates have ignorable subject effect as we have hoped for. On the other hand, it might also indicate that the proposed Bayesian model is not very sensitive to the presence of small subject effect in the data structure.

**Table 2:** Similar to Table 1, but this comparison is based on the second set of simulation studies (with subject effect)

		Logistic	DSS	*z*-test	Bayesian
		regression			
*γ*=0.01	FDR	0.0027	0	0.0034	0.0069
	mdFDR	0.0029	0	0.0036	0.008
	TPR	0.1953	0.0171	0.183	0.2494
	TPR_hypo	0.1948	0.0167	0.1918	0.2545
	TPR_hyper	0.1958	0.0176	0.171	0.2418
*γ*=0.05	FDR	0.0097	0	0.0113	0.014
	mdFDR	0.0097	0	0.0113	0.0141
	TPR	0.3554	0.0364	0.3235	0.4038
	TPR_hypo	0.3604	0.0375	0.3392	0.419
	TPR_hyper	0.3494	0.035	0.3044	0.3853
*γ*=0.1	FDR	0.0119	0	0.0142	0.0156
	mdFDR	0.0119	0	0.0142	0.0156
	TPR	0.5053	0.0668	0.4588	0.5745
	TPR_hypo	0.5021	0.0692	0.4637	0.5836
	TPR_hyper	0.509	0.0641	0.453	0.5637
*γ*=0.15	FDR	0.012	0	0.0147	0.0156
	mdFDR	0.012	0	0.0147	0.0156
	TPR	0.6385	0.1074	0.5939	0.7158
	TPR_hypo	0.6309	0.112	0.5905	0.7205
	TPR_hyper	0.6477	0.1016	0.5981	0.7101
*γ*=0.2	FDR	0.0116	0.0001	0.0146	0.0153
	mdFDR	0.0116	0.0001	0.0146	0.0153
	TPR	0.7546	0.1576	0.7203	0.8254
	TPR_hypo	0.7459	0.1612	0.7129	0.8287
	TPR_hyper	0.7659	0.1529	0.7298	0.821

## Real data analysis

In this section, we conduct both statistical analysis and bioinformatics analysis on a real dataset to compare the performance of difference procedures. The real dataset we analyze is the same CLL data [17] introduced at the beginning of the simulation section. Two CLL samples with matched covariates are selected to be compared with two control samples. There are 384,890 loci commonly mapped by these four samples. To reduce the dimension, we first remove loci that are either fully or non-methylated for all observed samples because there is no differential methylation associated with these loci. This reduces the dimension of the data to 324,126 loci. In other words, 60,764 loci are filtered out before we apply various procedures to detect DML.

### Statistical analysis

We apply to the real dataset the same four statistical procedures studied in the simulation studies. However, since the simulation studies show that the logistic regression approach always performs better than the *z* test and the DSS procedure, we focus on the comparison of the proposed Bayesian method with the logistic regression for the real data analysis in the main text while reporting the results for the *z* test and the DSS procedures in the Additional file 1. Since we do not know the actual FDR level of the proposed Bayesian method for the real data, we cannot control the FDR level of the other three procedures at the same level as the proposed Bayesian method. However, from the simulation studies we learn that the actual FDR level for the proposed method ranges from around 0.01 to 0.03. Therefore we consider various nominal FDR levels for the logistic regression method (and the *z* test and the DSS procedures) to match with this range. To be specific, we consider nominal FDR levels of 0.01, 0.02, 0.03 and 0.05. Table 3 gives the numbers of hypo- and hyper-methylated loci identified by the proposed method and by the logistic regression approach at the four different nominal FDR levels (the results for the *z* test and DSS procedure are given in Additional file 1: Table S6). As expected, the power of the logistic regression method increases with the nominal FDR level. Since the actual FDR of the proposed Bayesian approach never exceeds 0.022 for all our simulations settings, it is reasonable to compare its result with that of the logistic regression method at nominal level 0.02 or 0.03. Table 3 shows that the proposed Bayesian approach detects more DML than the logistic regression approach with nominal FDR levels no greater than 0.03. Specifically, the improvement is 28.9 *%* for hypo-methylated loci and 19.8 *%* for hyper-methylated loci when compared to the logistic regression model at level 0.02 and the improvement is 15.3 *%* and 9.2 *%* for hypo- and hyper-methylated loci respectively when the FDR level is set to be 0.03. This is consistent with our simulation studies: the proposed Bayesian method is more powerful than the logistic regression model when their FDR level is matched. When the nominal FDR level increases to 0.05 for the logistic regression approach, it detects slightly more DML than the proposed Bayesian method with only 2 *%* more hypo-methylated loci and 5 *%* more hyper-methylated loci. However, the cost is its increased false discovery rate. To examine this, we look at the detected DML disagreed by these two methods in Table 4.

**Table 3:** Numbers of hypo- and hyper-methylated loci identified by the proposed Bayesian method and logistic regression method for the real data. FDR is controlled at levels 0.01, 0.02, 0.03 and 0.05 for logistic regression

	Hypo-	Hyper-	
	methylation	methylation	
The proposed Bayesian method	31,328	21,270	
Logistic Regression: FDR controlled at 0.01	20,527	15,166	
Logistic Regression: FDR controlled at 0.02	24,309	17,758	
Logistic Regression: FDR controlled at 0.03	27,172	19,483	
Logistic Regression: FDR controlled at 0.05	31,863	22,419

**Table 4:** The numbers of DML disagreed by the proposed Bayesian method and the logistic regression method for the real data analysis. The nominal FDR levels of the logistic regression model are 0.01,0.02, 0.03 and 0.05. In the table, 0, 1 and 2 represent equal-, hypo- and hyper-methylated loci respectively

						Logistic regression										
		FDR 0.01				FDR 0.02				FDR 0.03				FDR 0.05		
		0	1	2		0	1	2		0	1	2		0	1	2
Bayesian method	0		42	41			512	368			1,397	852			3,795	2,245
	1	10,843		0		7,531		0		5,553		0		3,260		0
	2	6,145	0			3,880	0			2,639	0			1,096	0	

**Fig. 1:**
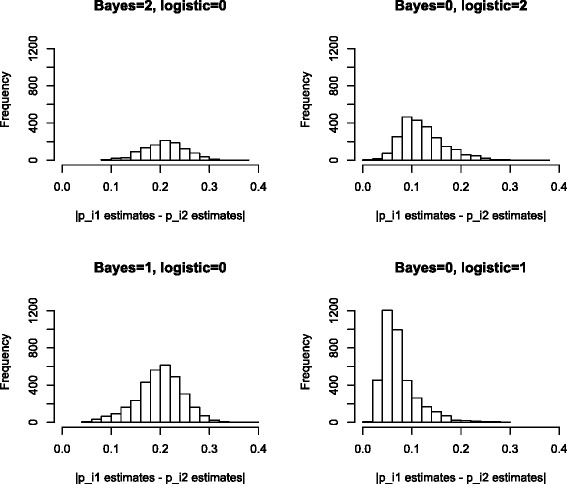
For the loci uniquely identified to be hypo-methylated loci (*group 1*) or hyper-methylated loci (*group 2*) by either the proposed Bayesian method or logistic regression when applied to the real data, four panels are histograms of absolute difference for methylation proportion estimates between the case and control samples. These four panels correspond to the counts in Table 4 when FDR level for logistic regression is controlled at 0.05 level

Table 4 shows that there are more DML (both hypo- and hyper-methylated loci) uniquely identified by the proposed Bayesian method than the logistic regression approach when the nominal FDR level of the logistic regression approach is no greater than 0.03. When this nominal level increases to 0.05, there are slightly more uniquely identified DML by the logistic regression method. We also notice that as the nominal FDR level of the logistic regression method increases, the number of the DML uniquely identified by the proposed Bayesian method is smaller, which may imply that the DML uniquely detected by the proposed Bayesian method can be verified by the logistic regression method at slightly higher nominal FDR level and hence are more likely to be true signals than false positives. On the other hand, the increasing number of DML uniquely identified by the logistic regression approach might just be the result of including more false positives. To see this better, we compare the histograms of the observed methylation proportion differences of the uniquely identified DML by either the proposed Bayesian approach or the logistic regression approach at various nominal FDR levels in Fig. 1 and Additional file 1: Figures S4, S5 and S6. The right panels of Fig. 1 and Additional file 1: Figures S4, S5 and S6 show that the DML uniquely identified by the logistic regression method at various nominal FDR levels mostly have small observed methylation proportion difference. If one follows the common practice of filtering out DML with methylation proportion difference smaller than 0.2 or 0.25 [9], then the majority of the DML uniquely identified by the logistic regression method will be filtered out for further study. On the other hand, the DML uniquely identified by the proposed Bayesian method tends to have large effect size with half of the loci having difference larger than 0.2 (see left panels of the above mentioned figures) and hence these DML are not only statistically significant but also biologically significant.

When it comes to statistically significant DML, it is more likely for a small-effect-size locus to be a false positive than one with large effect size. Therefore, we suspect that the DML uniquely identified by the proposed Bayesian method are more likely to be true signals while the ones uniquely identified by the logistic regression method at FDR level 0.05 are more likely to be false positives. A simulation study is conducted to verify this conjecture and the results are presented in Additional file 1: Table S7. This table shows that on average, there are more DML uniquely identified by the logistic regression method at level 0.05 than the Bayesian method. However, among these uniquely identified DML by the logistic regression method, 57.42 % are false positives compared to 13.86 % false positives among the uniquely identified DML by the proposed Bayesian method. On the other hand, 88.25 % of the unique DML by the Bayesian method have proportion difference larger than 0.2 while only 26.84 % of the extra DML detected by the logistic regression method have difference larger than 0.2. Interestingly, almost 40 % of the uniquely identified DML by the logistic regression at level 0.05 have effect size less than 0.1 and among these small-effect-size loci, 99.48 % are false positives. On the other hand, all the DML uniquely identified by the proposed Bayesian method have methylation proportion difference larger than 0.1. Therefore, we can conclude that the proposed Bayesian method detects more large-effect-size DML with lower proportion of false positives than by the logistic regression method at level 0.05 although we did not specify a nominal FDR level for our procedure. This comparison helps us to realize that our Bayesian approach has another advantage over the other three approaches in that it doesn’t depend on a pre-specified nominal FDR level while maintaining a small false discovery rate. In conclusion, the proposed Bayesian method is not only a powerful tool for identifying DML but it is also very reliable procedure and tends to pick up biologically significant DML missed by other procedures.

We also present the results of the *z* test and the DSS procedures in the Additional file 1. Comparing Additional file 1: Table S6 with Table 3, we can see that the ranking of the four procedures is about the same as seen in the simulation studies. The DSS is the most conservative procedure and the *z* test detects slightly fewer DML than the logistic regression model. Additional file 1: Figures S7, S8, S9 and S10 draw the Venn diagrams of the data analysis results of the four procedures at different nominal FDR levels (note that the result for the Bayesian approach doesn’t depend on the specification of the nominal FDR level) and for hypo- and hyper-methylated loci separately. By examining these Venn diagrams, we can see that all DML identified by the DSS are also identified by the proposed Bayesian method and the logistic regression method, which might imply that the DSS procedure is good for ranking the DML but it needs the correct null distribution to produce the right p-value for appropriate FDR control. The current default assumption of standard normal distribution produces very conservative *p*-values for the DSS procedure and need to be improved. The *z* test does not overlap with the proposed Bayesian method as well as the logistic regression method. The histograms of the observed methylation differences in Additional file 1: Figures S11, S12, S13 and S14 show that the majority of the DML identified by the *z* test but not by the proposed Bayesian method have observed methylation proportion differences smaller than 0.1.

### Bioinformatics analysis

To further compare the performance of the proposed Bayesian method and the logistic regression approach, we conduct some bioinformatics analysis. As we discuss in the introduction session, the differential methylation can influence the gene expression regulation. Therefore, a common next step after identifying the DML is to find the genes associated with the detected DML. Since multiple DML can associate with the same gene, identifying a larger number of DML does not guarantee identification of more associated genes. Therefore it is of interest to compare the number of genes uniquely identified by different methods. Table 5 gives the number of genes uniquely identified by either the proposed Bayesian method or the logistic regression approach or by both methods. It is very clear to see even in terms of genes, the proposed Bayesian method can detect more than the logistic regression methods at FDR level no greater than 0.03. We also notice that the number of commonly identified genes by both methods are increasing with the nominal FDR level of the logistic regression, which implies that the genes uniquely identified by the proposed Bayesian method can be verified by the logistic regression method at higher nominal FDR level. Here we want to focus on the genes that are uniquely identified by our proposed Bayesian method but missed by the logistic regression method even when we increase its nominal level to 0.05 (recall it is unlikely that the proposed Bayesian method has actual FDR level greater than 0.05). There were 129 genes with hyper-methylated CpGs and 365 genes with hypo-methylated CpGs that were found only by the proposed Bayesian method and not by the logistic regression method at FDR cut-off 0.05. A list of these genes are provided in Additional file 1: Tables S8 and S9. Further investigation of these genes indicates their biological relevance towards cancer regulation and proliferation. Uniquely identified genes with hypo-methylated CpGs consisted of genes associated with important functions like negative regulation cell death/apoptosis (genes include *BCL6, BAG1, CD27, G2E3, POU3F3, RASA1, AZU1, EGFR, EDNRB, MAPK81P1, MALT1, MCL1 and TGFBR1*), cell-cycle regulation (genes include *CDKN1B, CDKN2A and RUNX1*) and leukocyte activation (genes include *BCL6, BCL11A, BST2, RAB27A, AZU1, CBFB, MALT1 and NCR1*). Uniquely identified genes with hyper-methylated CpGs mostly consisted of homeobox genes (eg *HOXA1, HMBOX1*) and genes associated with regulation of transcription (eg. *NFATC4, TCF12, HMBOX1, HOXA1, SOX5, SIX4, ESR1*) or transcription silencing (eg. *YY1*). The proposed method identified hyper-methylated CpG 37 bp upstream to the TSS (Transcription Start loci) of *CASP7*, which is one of the key regulator genes in apoptosis execution. Overall, 37 genes were uniquely identified with hyper-methylated CpG and 102 genes with hypo-methylated CpGs within 1000bp proximity to their TSS.

**Table 5:** Number of genes associated with hypo- and hyper-methylated loci uniquely identified by the proposed Bayesian method or the logistic regression method, or identified by both methods. The nominal FDR levels of the logistic regression model are 0.01,0.02, 0.03 and 0.05. In the table, 0, 1 and 2 represent equal-, hypo- and hyper-methylated loci respectively

						Logistic regression										
		FDR 0.01				FDR 0.02				FDR 0.03				FDR 0.05		
		0	1	2		0	1	2		0	1	2		0	1	2
Bayesian method	0		1	3			36	52			82	132			224	391
	1	745	2,293	0		475	2,563	0		329	2,709	0		129	2,909	0
	2	1,379	0	4,043		920	0	4,502		648	0	4,774		365	0	5,057

Interesting, if we compare the proposed Bayesian method with the logistic regression method at FDR 0.03 for the bioinformatics analysis, it uniquely detects 200 more genes associated with hypo-methylated CpGs and 283 more genes associated with hyper-methylated CpGs. Among these extra uniquely identified genes by the proposed Bayesian method, many are directly associated with B-cells (or B-lymphocytes/leukocyte) and CLL cancer type. GO annotations like “Leukocyte/lymphocyte differentiation and activatio” (for *CD1D, CARD11, FOXP1, HDAC4, IRF4, NTRK1, SPN, SYK, SNCA, YWHAZ*) and “Wnt signalling” (for *AXIN2, FZD2, MACF1, NXN, SLC9A3R1, WNT3*) and “regulation cell proliferation” (for *PINX1, TGIF1, AXIN2, FOXJ1, IGFBP7, LDOC1, PTH1R, RUNX3, B4GALT7*) were enriched for genes uniquely identified by the proposed Bayesian method for hyper-methylation. Similarly, B-lymphocyte specific biological process like “Leukocyte activation” (for *BCL6, BCL11A, BST2, RAB27A, ULBP1, AZU1CBFB, HSH2D, HSH2D, ILI2B, MALT1, NCR1, YWHAZ*), and cancer development related like “negative regulation of cell death/apoptosi” (*BCL6, BAG1, CD27, G2E3, IHH, POU3F3, IHH, RASA1, AZU1, EDNRB, EGFR1, MCL1, TGFBR1 etc.*), negative regulation of cell differentiation (*BCL6, IHH, LMX1A, THY1, BMP4, RUNX1, NRP1, FOXA2, etc*), G1/S transition of cell cycle (*E2F6, CDKN1B, CDKN2A, EGFR, GFI1, SPDYA*), Cell migration (*LMX1A, EDNRB, KIF5C, IL12B, TGFR1*) were enriched for genes uniquely identified by the proposed Bayesian method for hypo-methylation. Note that these biologically relevant genes, although were not identified by the logistic regression method at FDR level 0.03, were verified when the FDR level increases to 0.05. This is in consistent with our earlier conclusion that the DML identified uniquely by the proposed Bayesian method is more likely to be true positives and can often be verified by the logistic regression method at the cost of increased FDR level.

## Conclusion and discussion

We propose a full Bayesian partition model for identifying differentially methylated loci under two conditions. It is well known that hypo-methylation and hyper-methylation plays different roles in gene regulations and have distinct biological meanings. Therefore it is important to differentiate these two types of differential methylation in the data analysis. Many existing methods including the logistic regression approach of Methylkit [9] and the empirical Bayes Wald test [10] only produce the binary output of a locus being differentially methylated or not. A common practice to further identify hypo- or hyper-methylated loci is based on the sign of the test statistics, which is an ad-hoc approach and ignores the uncertainty associated with the sign of the test statistics. Our proposed Bayesian partition model addresses the issue systematically by partitioning all loci into three groups: equal-, hypo- and hyper-methylated groups so that the final output of our analysis is more informative than just being differentially methylated or not. Compared to frequentist approaches such as logistic regression model of Methylkit [9], our Bayesian approach also has the advantage of borrowing strength across the loci and hence is more powerful for analyzing DNA methylation data with small sample sizes. Another advantage of the proposed Bayesian method is that one do not need to specify a nominal FDR level ahead of time, which sometimes can be a challenging job. Too small FDR level means shorter list of DML and too large FDR level can mean longer list of DML with more false positives. The proposed Bayesian model is shown by both simulation studies and real data analysis to have achieved higher power than other procedures while maintaining a low false discovery rate. It also tends to pick up biologically significant DML with large effect size missed by other methods. Therefore it is a both powerful and reliable procedure for identifying DML for single nucleotide resolution sequencing data.

Note our Bayesian model makes several assumptions such as parametric prior distributions and no subject effect for the methylation proportions within each condition. However our simulation studies try to mimic the real data as much as possible by generating the parameters from the empirical distribution of parameter estimates based on real data, which is not the prior distribution we assumed for the model. The good performance of our model show that our model is robust to the misspecification of the prior distribution. Furthermore, even when we generate data with subject effect on the methylation proportions, the proposed Bayesian model still performs similarly to the case without subject effect, which shows that our model is not very sensitive to the presence of small subject effect. However, it is important to address the issue of subject effect or biological variation on the methylation proportions for different biological samples within the same condition by modeling it correctly. This is one of our ongoing projects.

It is known that the methylation levels of adjacent CpG loci are correlated and hence it is important to consider the correlation structure of adjacent CpG loci. In this paper, our model assumes that the loci are conditional independent given parameters. Marginally, they are dependent because they share parameters. However,the correlation structure can also be modelled explicitly and this is one of our future work.

Although compared to other existing methods, our proposed method provides more accurate and reliable results, it is computationally more intensive. In terms of computing time, the proposed method takes approximately 42 hours to analyze 384,890 loci in a 2.80 GHz 4-core CPU, 16 GB memory environment while the logistic regression method, the DSS method and the z test take approximately 24, 5 and 3 minutes respectively in the same environment. Even though the computing time of our method is not infeasible in practice, it is desirable to improve the computing efficiency of our method to be closer to those of other methods, which is another future research direction for us.

## Additional file

Additional file 1
**Supplementary document.** This is a PDF document with three parts not shown in the paper. The first part is the derivation for the posterior distribution of ***I***, and the second and third parts are additional tables and figures. Tables contains the proportion of hypo- and hyper-methylated loci in the real data at different *γ* values, comparison in identification of the four procedures for simulation study with and without subject effect, relative power improvement of the proposed Bayesian method compared to three other methods for simulation study with and without subject effect, number of hypo- and hyper-methylated loci identified by DSS and *z* test, simulation study of the proportion of false positives in the list of uniquely identified loci by either the proposed Bayesian method or the logistic regression method at 0.05 FDR and list of genes with hypo- and hyper-methylated CpGs uniquely identified by the proposed Bayesian. Figures include that Figures S1 and S3 are histograms and normal QQ plots of the empirical Bayes Wald test statistics for one simulated data with and without subject effect, Figure S2 is histograms of *p* values for the empirical Bayes Wald test under the null hypothesis on one simulated data with and without subject effect, Figures S4, S5 and S6 are histograms of absolute difference for methylation proportion estimates between the case and control samples in the real data for the loci uniquely identified to be hypo- and hyper-methylated loci by either the proposed Bayesian and logistic regression at different FDR levels 0.01, 0.02, 0.03, Figure S7, S8, S9 and S10 are Venn diagrams of detected hypo- and hyper-methylated loci in real data analysis for four procedures with FDR controlled at 0.01, 0.02, 0.03 and 0.05, and Figures S11, S12, S13 and S14 are similar histograms with Figures S4, S5 and S6 but different comparison of the methods by either the proposed Bayesian and *z* test at different FDR levels 0.01, 0.02, 0.03 and 0.05. (PDF 3160 kb)
