# Supplementary material for: A full Bayesian partition model for identifying hypo- and hyper-methylated loci from single nucleotide resolution sequencing data
Source: BMC Bioinformatics. 2016 Jan 11;17(Suppl 1):7. doi: 10.1186/s12859-015-0850-3 (PMC4895387; doi:10.1186/s12859-015-0850-3)
Supplement: Additional file 1 — Supplementary document. This is a PDF document with three parts not shown in the paper. The first part is the derivation for the posterior distribution of I, and the second and third parts are additional tables and figures. Tables contains the proportion of hypo- and hyper-methylated loci in the real data at different γ values, comparison in identification of the four procedures for simulation study with and without subject effect, relative power improvement of the proposed Bayesian method compared to three other methods for simulation study with and without subject effect, number of hypo- and hyper-methylated loci identified by DSS and z test, simulation study of the proportion of false positives in the list of uniquely identified loci by either the proposed Bayesian method or the logistic regression method at 0.05 FDR and list of genes with hypo- and hyper-methylated CpGs uniquely identified by the proposed Bayesian. Figures include that Figures S1 and S3 are histograms and normal QQ plots of the empirical Bayes Wald test statistics for one simulated data with and without subject effect, Figure S2 is histograms of p values for the empirical Bayes Wald test under the null hypothesis on one simulated data with and without subject effect, Figures S4, S5 and S6 are histograms of absolute difference for methylation proportion estimates between the case and control samples in the real data for the loci uniquely identified to be hypo- and hyper-methylated loci by either the proposed Bayesian and logistic regression at different FDR levels 0.01, 0.02, 0.03, Figure S7, S8, S9 and S10 are Venn diagrams of detected hypo- and hyper-methylated loci in real data analysis for four procedures with FDR controlled at 0.01, 0.02, 0.03 and 0.05, and Figures S11, S12, S13 and S14 are similar histograms with Figures S4, S5 and S6 but different comparison of the methods by either the proposed Bayesian and z test at different FDR levels 0.01, 0.02, 0.03 and 0.05. (PDF 3160 kb) [file 12859_2015_850_MOESM1_ESM.pdf]

A Full Bayesian Partition Model for Identifying Differentially Methylated Loci from Single Nucleotide Resolution Sequencing Data

Henan Wang, Chong He, Garima Kushwaha, Dong Xu, Jing Qiu

November 6, 2015

To make inference about the membership of each loci along the whole genome, the derivation for the posterior distribution of $\boldsymbol{I}$ can be given by

$$
\begin{aligned}
p(\boldsymbol{I}|\boldsymbol{C},\boldsymbol{M}) &= \frac{p(\boldsymbol{C},\boldsymbol{M}|\boldsymbol{I})p(\boldsymbol{I})}{\int p(\boldsymbol{C},\boldsymbol{M}|\boldsymbol{I})p(\boldsymbol{I})d\boldsymbol{I}} \\
&= \frac{p(\boldsymbol{C}|\boldsymbol{I})p(\boldsymbol{M}|\boldsymbol{C},\boldsymbol{I})p(\boldsymbol{I})}{\int p(\boldsymbol{C}|\boldsymbol{I})p(\boldsymbol{M}|\boldsymbol{C},\boldsymbol{I})p(\boldsymbol{I})d\boldsymbol{I}} \\
&= \frac{p(\boldsymbol{M}|\boldsymbol{C},\boldsymbol{I})P(\boldsymbol{I})}{\int p(\boldsymbol{M}|\boldsymbol{C},\boldsymbol{I})p(\boldsymbol{I})d\boldsymbol{I}} \text{ (since } \boldsymbol{C} \text{ and } \boldsymbol{I} \text{ are independent, } p(\boldsymbol{C}|\boldsymbol{I}) = p(\boldsymbol{C})) \\
&= \frac{p(\boldsymbol{M_0}|\boldsymbol{C},\boldsymbol{I})p(\boldsymbol{M_1}|\boldsymbol{C},\boldsymbol{I})p(\boldsymbol{M_2}|\boldsymbol{C},\boldsymbol{I})p(\boldsymbol{I})}{\int p(\boldsymbol{M}|\boldsymbol{C},\boldsymbol{I})p(\boldsymbol{I})d\boldsymbol{I}} \text{ (assume } \boldsymbol{M_0}, \boldsymbol{M_1} \text{ and } \boldsymbol{M_2} \text{ are independent)} \\
&= \frac{p(\boldsymbol{M_0}|\boldsymbol{C_0},\boldsymbol{I})p(\boldsymbol{M_1}|\boldsymbol{C_1},\boldsymbol{I})p(\boldsymbol{M_2}|\boldsymbol{C_2},\boldsymbol{I})p(\boldsymbol{I})}{\int p(\boldsymbol{M}|\boldsymbol{C},\boldsymbol{I})p(\boldsymbol{I})d\boldsymbol{I}} \\
&\propto p(\boldsymbol{M_0}|\boldsymbol{C_0},\boldsymbol{I})p(\boldsymbol{M_1}|\boldsymbol{C_1},\boldsymbol{I})p(\boldsymbol{M_2}|\boldsymbol{C_2},\boldsymbol{I})p(\boldsymbol{I})
\end{aligned}
$$

Tables

Table S1 – The proportion of hypo- and hyper-methylated loci in the real data at different γ values.

	Hypo-methylated	Hyper-methylated
$\gamma = 0.01$	43.28%	29.44%
$\gamma = 0.05$	20.58%	16.79%
$\gamma = 0.1$	12.12%	10.28%
$\gamma = 0.15$	8.28%	6.77%
$\gamma = 0.2$	5.9%	4.6%

Table S2 – Comparison in identification of the four procedures (logistic regression, DSS, z-test and the proposed Bayesian) at different γ values for the simulation studies without subject effect. Results are averaged over 100 replications of 20,000 CpG loci with two samples under each condition.

	True I	I from logistic			I from DSS			I from z test			I from Bayesian		
		0	1	2	0	1	2	0	1	2	0	1	2
$\gamma = 0.01$	0	5231.04	7.73	6.13	5244.83	0	0.07	5228.42	10.64	5.84	5210.42	21.17	13.31
	1	6640.29	1831.91	0.94	7835.93	637.19	0.02	6668.5	1804.05	0.59	6270.51	2199.77	2.86
	2	4933.68	0.69	1347.59	5683.67	0	598.29	5094.69	0.56	1186.71	4725.64	4.15	1552.17
$\gamma = 0.05$	0	11718	28.83	22.22	11768.62	0.17	0.26	11715.12	34.17	19.76	11705.7	37.2	26.15
	1	2743.55	1769.78	0.15	3795.98	717.5	0	2846.05	1667.36	0.07	2594.19	1918.86	0.43
	2	2309.26	0.22	1407.99	3058.6	0	658.87	2480.87	0.15	1236.45	2262.63	0.68	1454.16
$\gamma = 0.1$	0	14851.26	33.19	24.97	14908.74	0.27	0.41	14846.17	40.58	22.67	14844.66	37.75	27.01
	1	1273.35	1428.39	0	1970.2	785.54	0	1374.93	1380.81	0	1135.53	1620.18	0.03
	2	1069.24	0.01	1265.59	1626.9	0	707.94	1188.76	0	1146.08	1015.18	0.02	1319.64
$\gamma = 0.15$	0	16489.19	27.29	21.85	16537.6	0.29	0.44	16482.05	36.29	19.99	16483.35	31.41	23.57
	1	643.51	1260.08	0	1100.23	803.36	0	709.99	1193.6	0	523.88	1370.71	0
	2	501.82	0	1056.26	857.36	0	700.72	568.3	0	989.78	449.84	0	1108.24
$\gamma = 0.2$	0	17532.55	20.88	17.66	17570.3	0.3	0.49	17523.16	29.63	18.3	17527.48	25.41	18.2
	1	313.79	1055.4	0	598.59	770.6	0	352.99	1016.2	0	239.25	1129.94	0
	2	228.03	0	831.69	433.44	0	626.28	260.05	0	799.67	194.67	0	865.05

Table S3 – The relative power improvement of the proposed Bayesian method compared to three other methods (logistic regression, z-test and DSS) in terms of TPR, TPR_hypo and TPR_hyper at different γ values for the simulation studies without subject effect. Results are averaged over 100 replications of 20,000 CpG loci with two samples under each condition.

		logistic	z test	DSS
$\gamma = 0.01$	TPR	18.16%	25.64%	204.2%
	TPR_hypo	20.08%	21.94%	245.2%
	TPR_hyper	15.18%	30.8%	159.4%
$\gamma = 0.05$	TPR	6.17%	16.19%	145.1%
	TPR_hypo	8.42%	15.08%	167.4%
	TPR_hyper	3.28%	17.61%	120.7%
$\gamma = 0.1$	TPR	6.98%	16.34%	96.85%
	TPR_hypo	9.29%	17.34%	106.3%
	TPR_hyper	4.27%	15.14%	86.41%
$\gamma = 0.15$	TPR	7.02%	13.54%	64.82%
	TPR_hypo	8.78%	14.84%	70.62%
	TPR_hyper	4.92%	11.97%	58.16%
$\gamma = 0.2$	TPR	5.72%	9.86%	42.82%
	TPR_hypo	7.06%	11.19%	46.63%
	TPR_hyper	4.01%	8.18%	38.13%

Table S4 – Similar to Table S2, but this is the result for the simulation studies with subject effect.

	True I	I from logistic			I from DSS			I from z test			I from Bayesian		
		0	1	2	0	1	2	0	1	2	0	1	2
$\gamma = 0.01$	0	5237.13	4.67	3.1	5244.9	0	0	5235.63	6	3.27	5219.4	16.04	9.46
	1	6822.06	1650.74	0.34	8331.7	141.44	0	6847.71	1625.25	0.18	6314.54	2157.04	1.56
	2	5051.41	0.24	1230.31	6171.26	0	110.7	5207.47	0.2	1074.29	4760.34	2.56	1519.06
$\gamma = 0.05$	0	11740.41	16.67	11.97	11769.05	0	0	11738.63	18.79	11.63	11721.98	28.38	18.69
	1	2886.83	1626.63	0.02	4344	169.48	0	2982.41	1531.07	0	2622.28	1891.06	0.15
	2	2418.57	0.02	1298.88	3587.44	0	130.03	2585.75	0.02	1131.7	2284.87	0.23	1432.37
$\gamma = 0.1$	0	14878.56	18.11	12.75	14909.42	0	0	14875.87	20.96	12.59	14863.17	26.46	19.79
	1	1372.06	1383.68	0	2565.07	190.67	0	1477.79	1277.95	0	1147.56	1608.17	0.01
	2	1146.35	0	1188.49	2185.25	0	149.59	1277.16	0	1057.68	1018.64	0	1316.2
$\gamma = 0.15$	0	16511.59	15.88	10.86	16538.33	0	0	16507.75	19.81	10.77	16499.03	23.17	16.13
	1	702.6	1200.99	0	1690.3	213.29	0	779.57	1124.02	0	532.09	1371.5	0
	2	548.95	0	1009.13	1399.71	0	158.37	626.26	0	931.82	451.74	0	1106.34
$\gamma = 0.2$	0	17549.52	12.7	8.87	17571.07	0	0.02	17545.16	16.55	9.38	17539.91	18.64	12.54
	1	347.95	1021.24	0	1148.5	220.69	0	393.09	976.1	0	234.5	1134.69	0
	2	248.11	0	811.61	897.69	0	162.03	286.34	0	773.38	189.67	0	870.05

Table S5 – Similar to Table S3, but this relative power improvement is for the simulation studies with subject effect.

		logistic	z test	DSS
$\gamma = 0.01$	TPR	27.71%	36.31%	1359.59%
	TPR_hypo	30.67%	32.72%	1425.06%
	TPR_hyper	23.47%	41.4%	1272.23%
$\gamma = 0.05$	TPR	13.61%	24.82%	1009.75%
	TPR_hypo	16.26%	23.51%	1015.8%
	TPR_hyper	10.28%	26.57%	1001.57%
$\gamma = 0.1$	TPR	13.69%	25.21%	759.45%
	TPR_hypo	16.22%	25.84%	743.43%
	TPR_hyper	10.75%	24.44%	779.87%
$\gamma = 0.15$	TPR	12.11%	20.53%	566.7%
	TPR_hypo	14.2%	22.02%	543.02%
	TPR_hyper	9.63%	18.73%	598.6%
$\gamma = 0.2$	TPR	9.38%	14.59%	423.8%
	TPR_hypo	11.11%	16.25%	414.16%
	TPR_hyper	7.2%	12.5%	436.97%

Table S6 – Numbers of hypo- and hyper-methylated loci identified by DSS and z-test method for the real data. FDR is controlled at levels 0.01, 0.02, 0.03 and 0.05 for both methods.

	hypo-methylation	hyper-methylation
DSS: FDR controlled at 0.01	4,066	2,814
DSS: FDR controlled at 0.02	4,801	3,258
DSS: FDR controlled at 0.03	5,316	3,636
DSS: FDR controlled at 0.05	6,119	4,142
z-test: FDR controlled at 0.01	20,365	13,126
z-test: FDR controlled at 0.02	24,300	15,541
z-test: FDR controlled at 0.03	27,390	17,373
z-test: FDR controlled at 0.05	32,111	20,070

Table S7 – Summary of simulation studies to study the proportions of false positives in the list of uniquely identified loci by either the proposed Bayesian method or the logistic regression method at 0.05 FDR level (the results are averaged over 100 simulation datasets with subject effect when γ is 0.2). Here "total" indicates the total number of uniquely identified loci by either method and "total with difference >0.2" indicates the number of loci uniquely identified by either method with methylation proportion difference greater than 0.2. Similarly, we also check situations when the methylation proportion difference is less or equal than 0.2, and less than 0.1 respectively.

	Logistic regression	The proposed Bayesian
total	115.21	28.52
proportion of false positives (FPs) among uniquely identified DML	57.42%	13.86%
total with difference >0.2	30.92(26.84%)	25.17(88.25%)
proportion of FPs among the uniquely identified DML with difference >0.2	7.44%	11.14%
proportion of FPs among the uniquely identified DML with difference <=0.2	75.8%	33.73%
total with difference <0.1	45.17(39.21%)	0(0%)
proportion of FPs among the uniquely identified DML with difference <0.1	99.48%	0

Table S8 – List of genes with hyper-methylated CpGs uniquely identified by the proposed Bayesian model.

AAAS	*CENPV*	*FGF22*	*LIMD2*	*NFATC4*	*RP4-738P15.1*	*TMCC1*
AC009473.1	*CGB7*	*GALE*	*LINC00299*	*NRK*	*RRAGA*	*TMEM181*
ADCK2	*CHRM5*	*GPER1*	*LMNB2*	*ODC1*	*RTN1*	*TSPAN17*
AFF4	*CHRNA1*	*GPR113*	*LPCAT4*	*OR7E91P*	*RTP4*	*TUSC5*
AP1M2	*CKMT2-AS1*	*GRIA3*	*LUZP2*	*OTUD7A*	*SERPINB6*	*UBA52*
ARF5	*CLIP2*	*HIF1AN*	*MAN1C1*	*PDE11A*	*SH3BGRL3*	*UBL4B*
ARFGEF2	*CNKSR3*	*HIST1H2BO*	*MARCO*	*PINX1*	*SIRT6*	*URI1*
ASPSCR1	*CREBRF*	*HMBOX1*	*MDH1B*	*PIP5KL1*	*SIX4*	*WDR89*
ATE1	*CST7*	*HMHA1*	*MIR326*	*PLEKHA7*	*SLC29A1*	*WNT3*
AZI1	*CYB5A*	*HOXA1*	*MIR3659*	*PLEKHH2*	*SLC38A11*	*YWHAZ*
B4GALT7	*DFFB*	*HPCA*	*MIR4664*	*POLR3GL*	*SLFN11*	*YY1*
C10orf88	*DNAJB1*	*HPN-AS1*	*MIR4672*	*POMT1*	*SNRK*	*ZBP1*
C16orf13	*DNAJC28*	*IFNAR2*	*MIR499A*	*PRDX5*	*SOX5*	*ZNF267*
C16orf80	*EPHX3*	*IMP4*	*MIR6075*	*PRMT6*	*SPIRE2*	*ZNF277*
C19orf59	*ESAM*	*INSRR*	*MIR6743*	*RANGAP1*	*STK17A*	*ZNF555*
C1orf50	*ESR1*	*KCTD7*	*MIR6848*	*RBM47*	*STX16*	
CACNA1G	*FAM115A*	*KLK14*	*MKNK1-AS1*	*RBMXL2*	*TBC1D1*	
CASP7	*FAM205A*	*LARP1*	*MX2*	*RNF4*	*TCF12*	
CECR3	*FEZ1*	*LDHD*	*MYLK3*	*RP11-31F15.2*	*TGIF1*	

Table S9 – List of genes with hypo-methylated CpGs uniquely identified by the proposed Bayesian model.

ABTB2	CAPN9	FAM218A	LOC100506422	NXT1	RGR	SYT3
AC009473.1	CAST	FAM83D	LOC101928604	OAF	RNF138	TAS1R1
AC010975.1	CBFB	FAM83E	LOC101929217	OCIAD1	RNF139	TBCA
AC097662.2	CCDC167	FCN1	LOC101929528	OR11L1	RNF165	TCTEX1D4
ACOXL	CCDC68	FOXI2	LOC284023	OR13G1	RNLS	TDRD9
ACSM5	CCL16	FOXJ3	LOC440461	OR2AG1	RP11-421P23.1	TEAD3
ADAMTS16	CD27	FZD5	LRCH3	OR56A3	RP11-569G13.2	TECRL
ADAMTSL1	CD47	G2E3	LRIT1	OR7E91P	RP11-666I19.2	TEKT4
ADD2	CDKN1B	GALC	LRPAP1	OSCAR	RP11-807H7.1	TFAP2A-AS1
AGFG2	CDKN2A	GK	LRRC56	P4HB	RP11-857B24.5	TGFBR1
AGO3	CELSR2	GLIS3	LZTS1-AS1	PALLD	RP11-909B2.1	THOC1
AJUBA	CES3	GML	MALT1	PAOX	RP5-1180C10.2	THY1
AK2	CHIAP2	GNAL	MAN2B2	PCDHAC1	RPL6	TIPRL
AKAP9	CNKSR1	GNL3L	MAP4	PCDHB11	RRAGD	TMEM14C
AKTIP	CNOT3	GPR12	MAPK8IP1	PCDHGB2	RUNX1	TMEM180
ALDH18A1	CNPY3	GPR137C	MARCKS	PCSK2	S100A11	TMEM41B
ALG5	COCH	GPR156	MC5R	PELO	SAG	TRAF3IP1
ALOX5AP	COL18A1-AS2	GPR6	MCL1	PGS1	SAMD12-AS1	TRAM2
ALPP	CPEB1	GPR62	MEGF11	PHOSPHO1	SAMD9	TRIM8
AMH	CSAD	GRIA2	MEP1A	PHYHIP	SCARB2	TRPC4
ANAPC1	CSRNP1	GS1-259H13.2	MGC34796	PIGB	SCYL3	TRPM5
ANO2	CSTF2	GXYLT2	MIR10B	PIH1D3	SEMA5B	TUBGCP5
AP1AR	CUL4B	HDAC10	MIR340	PKD1L2	SERPINA7	TUSC1
AP5S1	CYP4F2	HIST3H2A	MIR365A	PLA2G4C	SGSM1	UBE2B
ARHGAP20	DAW1	HMGA1	MIR3688-2	PLD4	SH2B1	UBP1
ARHGDIB	DBNDD1	HSPA4	MIR3935	PLEKHA1	SHC2	ULK4
ATP5H	DBP	HSPA8	MIR4533	POU3F3	SKOR1	UNKL
ATP7B	DBX1	HSPB3	MIR4649	PPCDC	SLC13A1	UQCRFS1
AZU1	DCAF7	HTR1B	MIR4694	PPM1A	SLC25A20	UQCRHL
BAG1	DDX49	INTS5	MIR5091	PRICKLE4	SLC25A51	USP46
BAHD1	DIAPH1	IRX5	MIR548AS	PRKG1	SLC27A6	VIPR1
BCL11A	DLST	ISX	MIR548I1	PRMT1	SLC28A3	VSTM2B
BCL6	DLX2	ITGA8	MIR6795	PRR14L	SLC29A1	WBP11P1
BEAN1	DNAJC1	ITPRIPL2	MIR6867	PRSS22	SLC37A3	WDYHV1
BID	DNMT3B	IWS1	MKLN1	PRSS38	SLC46A1	WNT16
BMP4	DPEP2	KCNIP3	MKRN1	PSIP1	SLC4A8	YWHAB
BOC	DPH2	KIAA1024L	MRPL12	PSMA3	SLC6A12	ZFP36
BPIFC	DPP10	KLHDC10	MSI1	PSMC3	SMCO4	ZNF181
BRSK1	DPY19L2	KLK5	MTHFD2	PTAFR	SMKR1	ZNF184
BST2	DSP	LILRB2	MVD	PTBP3	SNX22	ZNF236
C10orf126	DUOXA1	LINC00442	NASP	PTPMT1	SPATA41	ZNF488
C10orf95	EDNRB	LINC00535	NBPF23P	PVRL1	SPDYA	ZNF496
C12orf45	EFR3B	LINC00644	NCF4	RAB27A	SRSF12	ZNF550
C1orf234	EGFLAM-AS2	LINC00700	NCR1	RAD54L2	SRSF4	ZNF606
C20orf27	EGFR	LINC00707	NDP	RAP1GAP2	SSNA1	ZNF718
C2orf50	EIF5	LINC00896	NDUFA11	RAP2B	ST6GALNAC6	ZNF768
C6orf132	EPB41L4A	LINC01003	NECAB2	RASA1	STAP2	ZNF99
C8B	ETFA	LINC01007	NFATC3	RASA4CP	STK25	
C8orf48	FAM118A	LINC01056	NOX1	RASGRP1	STMN2	
C8orf86	FAM126A	LINC01116	NR5A2	RAX2	STRA6	
CAMK1	FAM184B	LIPG	NRF1	RBM20	SUDS3	
CANT1	FAM213A	LMNB2	NRP1	RGCC	SYCE1L	
CAPN10-AS1	FAM215A	LOC100268168	NTN4	RGPD3	SYNJ1	

Figures

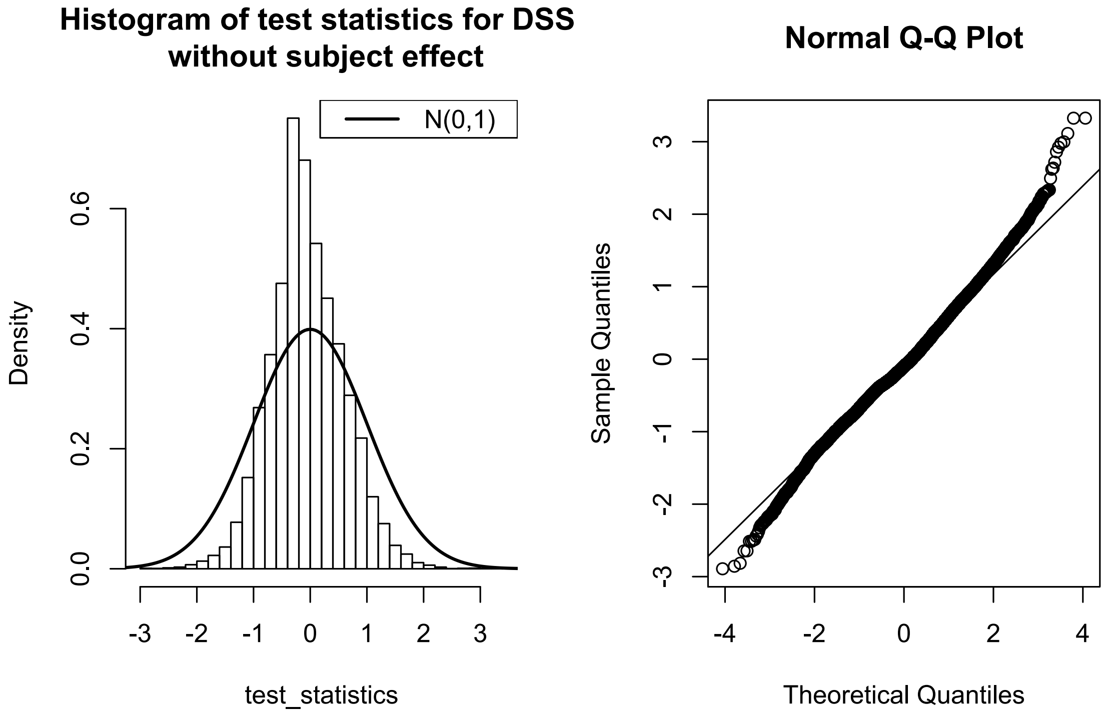

Figure S1 – Left panel: the histogram of the empirical Bayes Wald test statistics of the DSS procedure from one simulated data without subject effect compared with the standard normal curve. Right panel: the normal QQ plot of the empirical Bayesian Wald test statistics of one simulated data without subject effect.

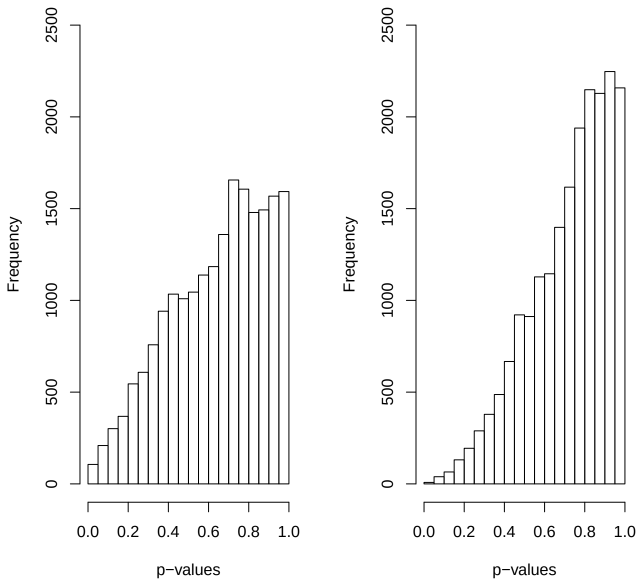

Figure S2 – Histograms of p-values for the empirical Bayes Wald test on one dataset simulated under the null hypothesis (i.e. all 20,000 CpG loci are simulated as equal-methylated). Left panel is for the first set of simulation studies (without subject effect) and right panel is for the second set of simulation studies (with subject effect).

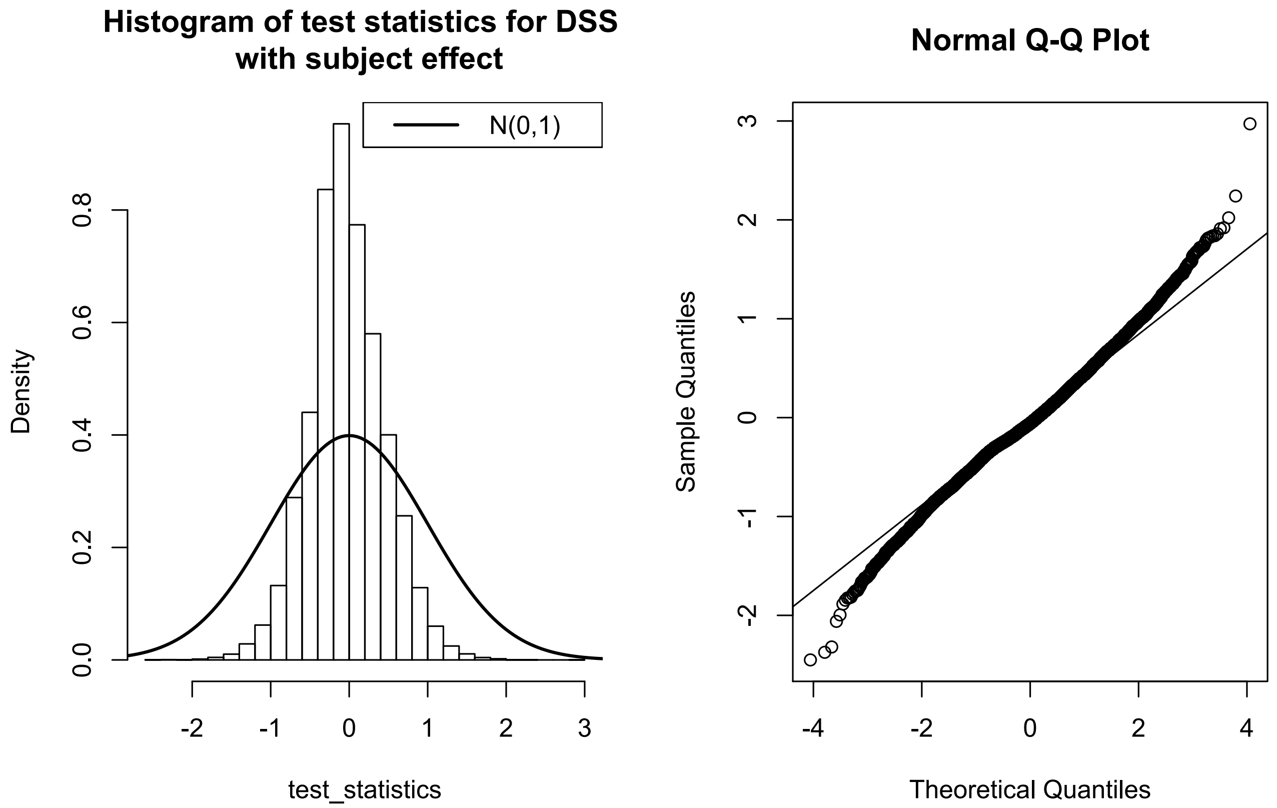

Figure S3 – Similar to Figure S1, except that this is for the simulation studies with subject effect.

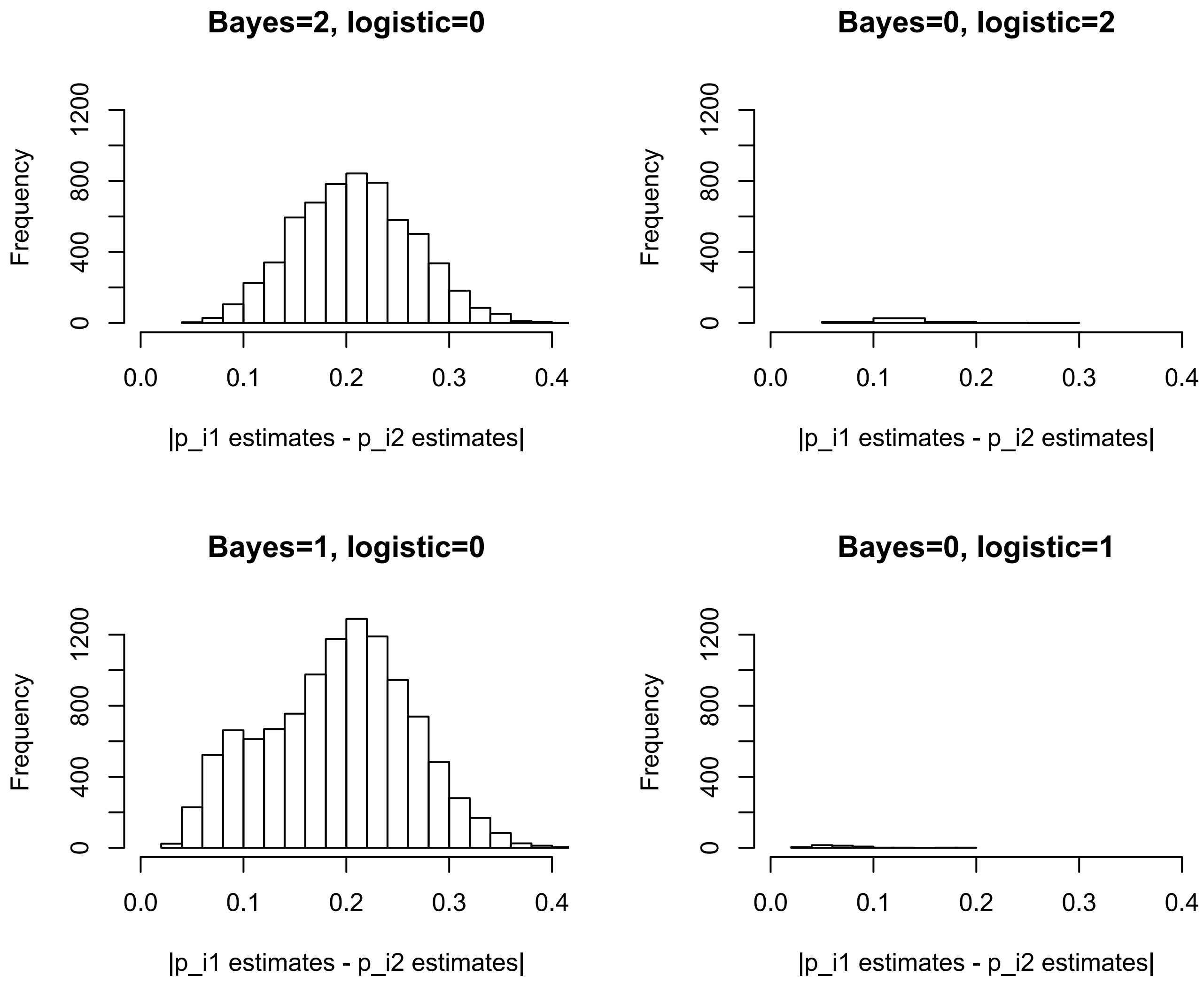

Figure S4 – For the loci uniquely identified to be hypo-methylated loci (group 1) or hyper-methylated loci (group 2) by either the proposed Bayesian method or logistic regression when applied to the real data, four panels are histograms of absolute difference for methylation proportion estimates between the case and control samples. These four panels correspond to the counts in Table 4 when FDR level for logistic regression is controlled at 0.01 level.

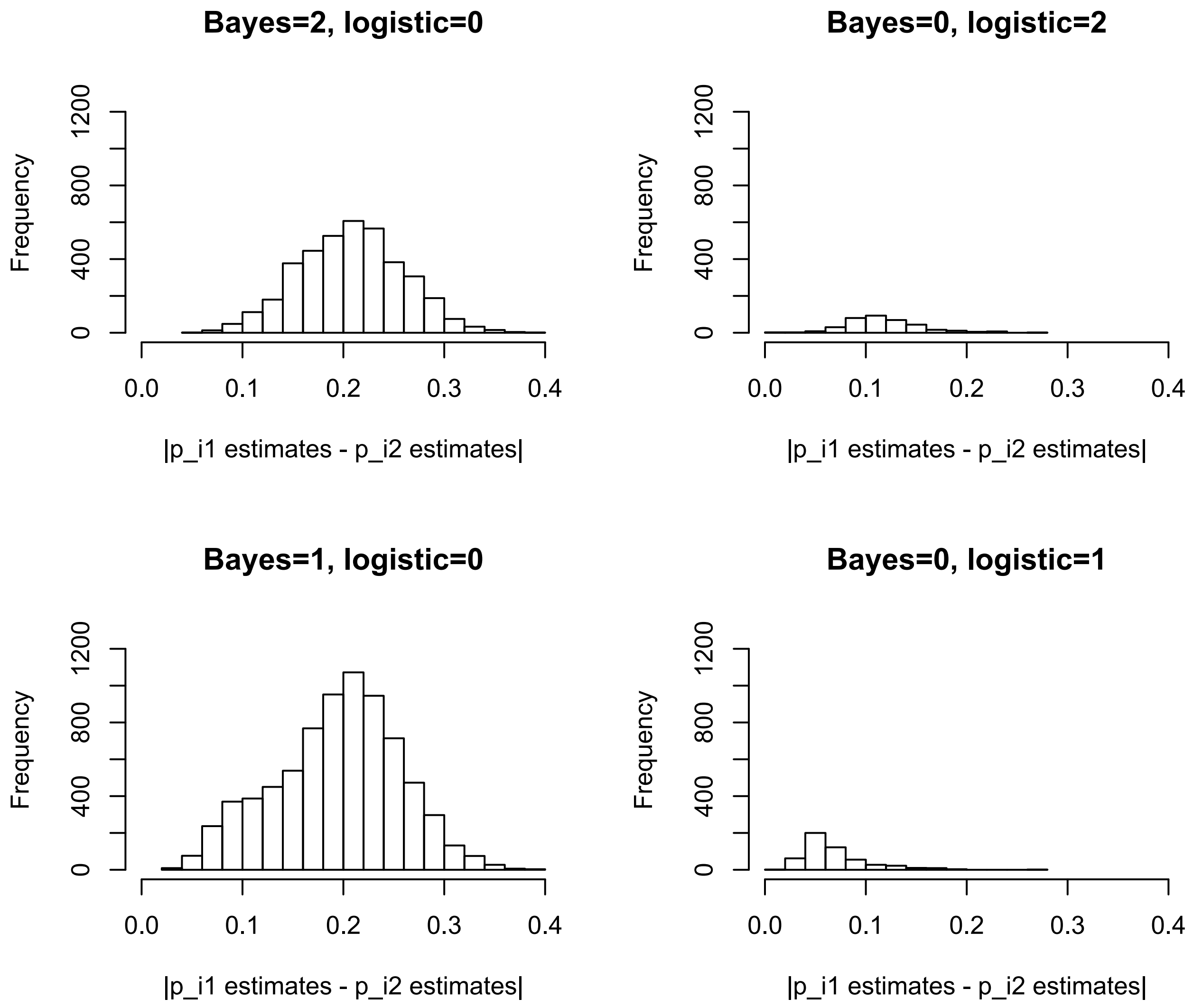

Figure S5 – Similar to Figure S4, except that FDR level for logistic regression is controlled at 0.02.

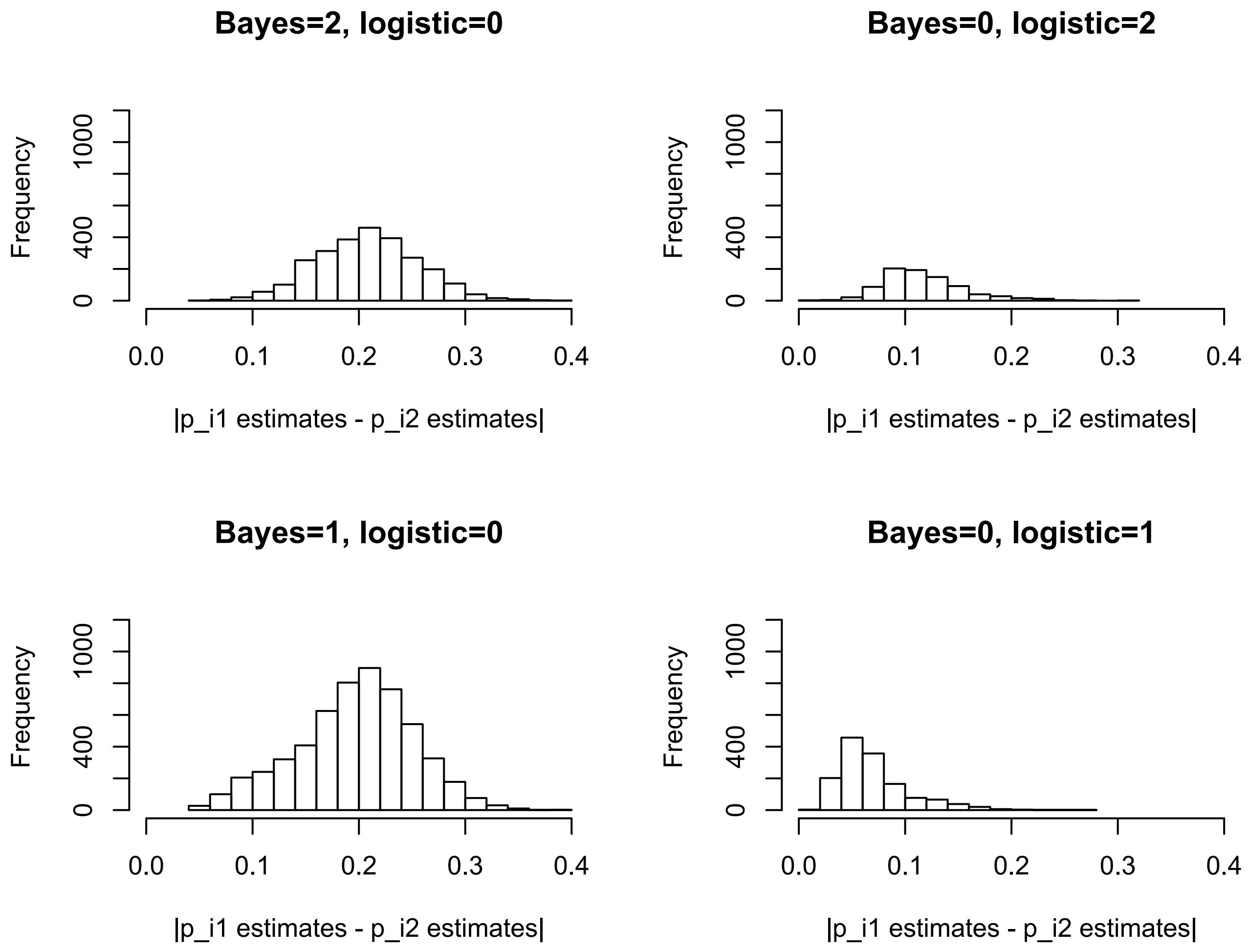

Figure S6 – Similar to Figure S4, except that FDR level for logistic regression is controlled at 0.03.

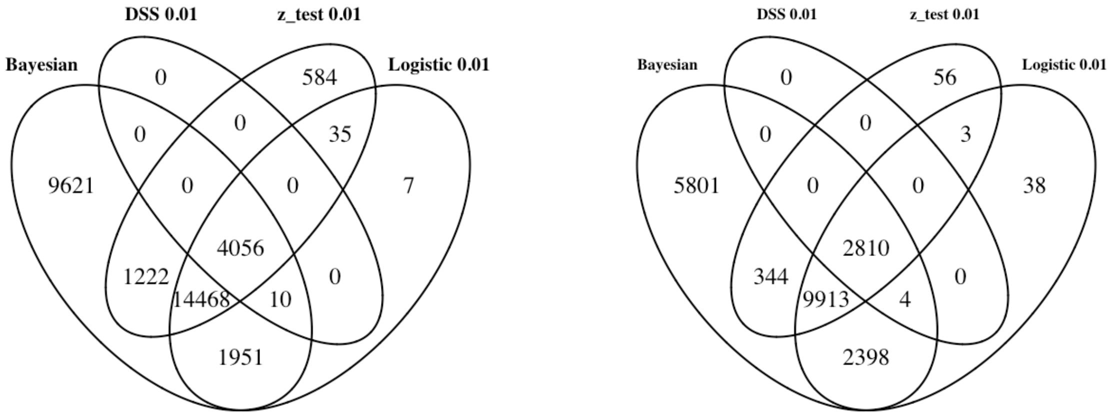

(a) Hypo-methylated

(b) Hyper-methylated

Figure S7 – Venn diagram of detected hypo- and hyper-methylated loci in real data analysis. FDR for logistic regression, DSS and z test is controlled at 0.01 level.

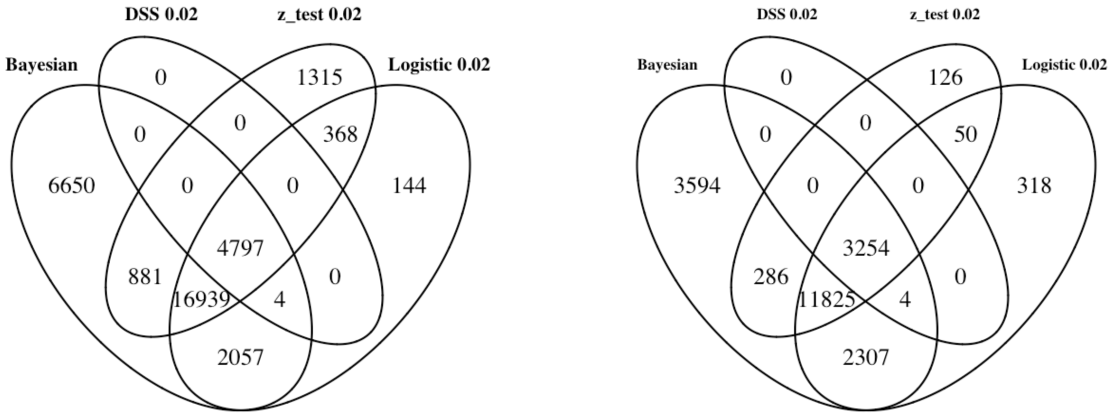

(a) Hypo-methylated

(b) Hyper-methylated

Figure S8 – Venn diagram of detected hypo- and hyper-methylated loci in real data analysis. FDR for logistic regression, DSS and z test is controlled at 0.02 level.

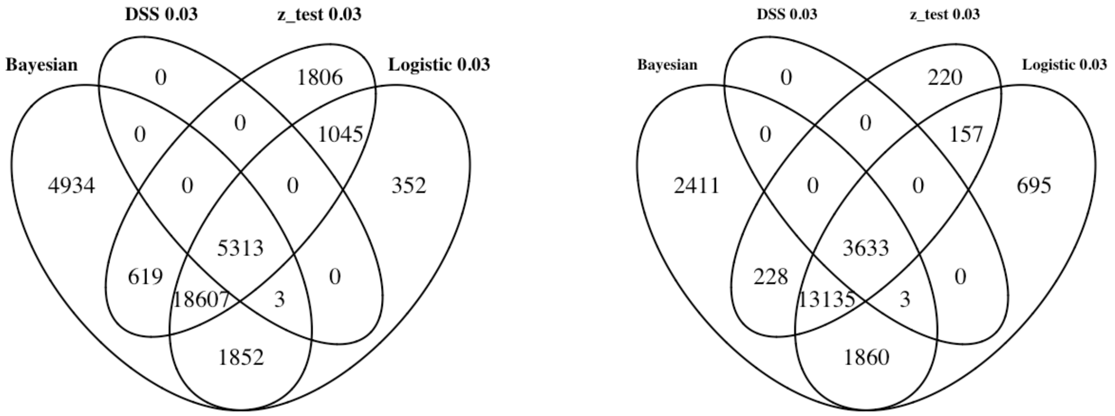

(a) Hypo-methylated

(b) Hyper-methylated

Figure S9 – Venn diagram of detected hypo- and hyper-methylated loci in real data analysis. FDR for logistic regression, DSS and z test is controlled at 0.03 level.

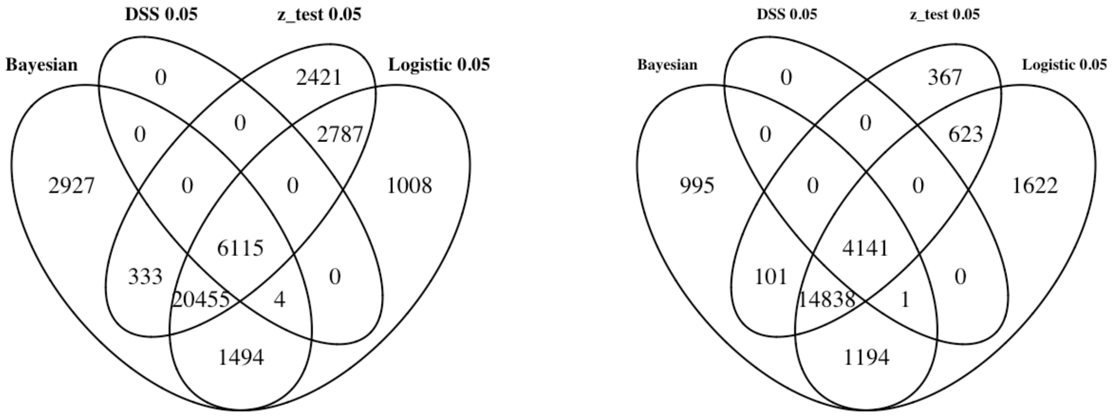

(a) Hypo-methylated

(b) Hyper-methylated

Figure S10 – Venn diagram of detected hypo- and hyper-methylated loci in real data analysis. FDR for logistic regression, DSS and z test is controlled at 0.05 level.

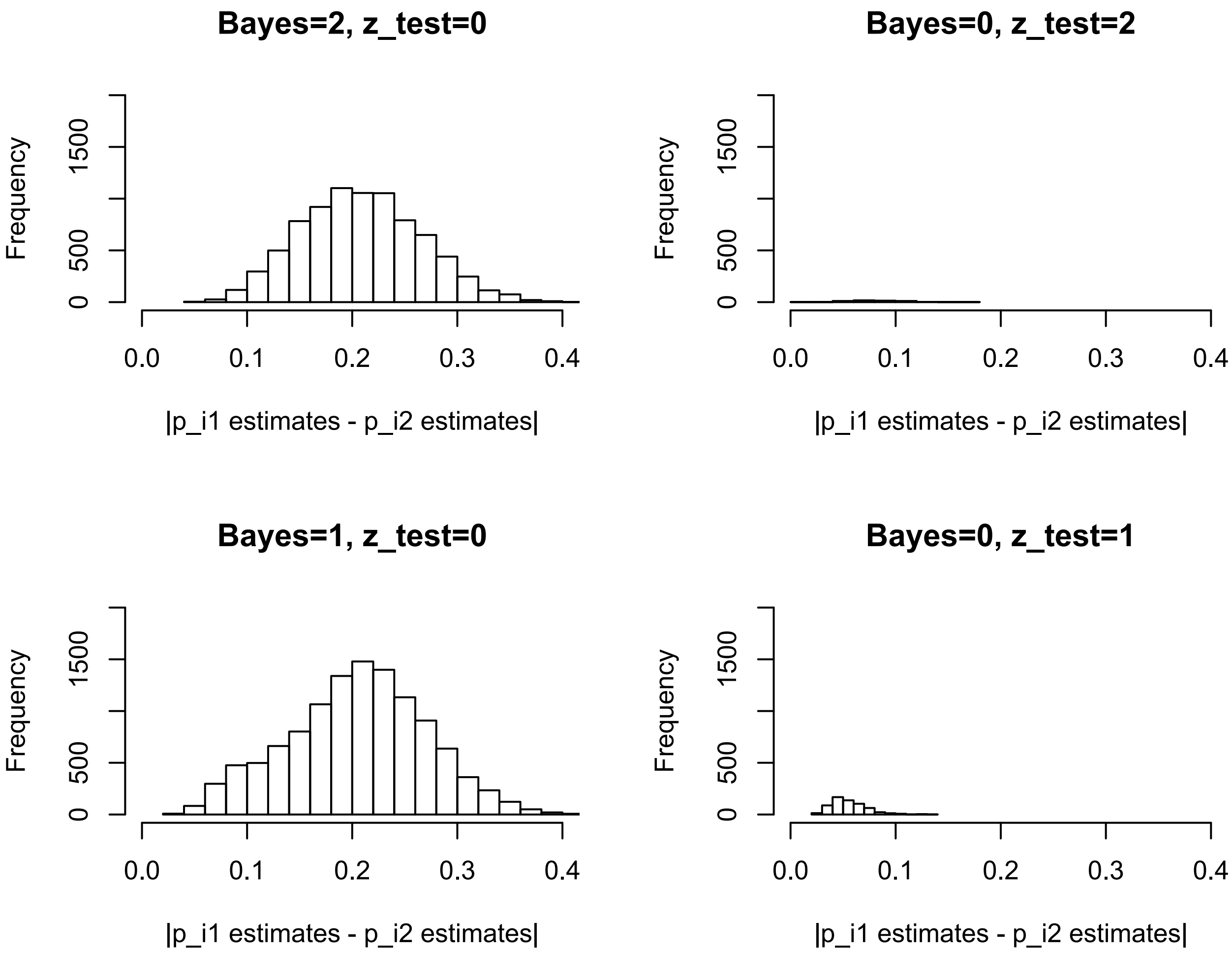

Figure S11 – For the loci uniquely identified to be hypo-methylated loci (group 1) or hyper-methylated loci (group 2) by either the proposed Bayesian method or z test when applied to the real data, four panels are histograms of absolute difference for methylation proportion estimates between the case and control samples. FDR level for z test is controlled at 0.01.

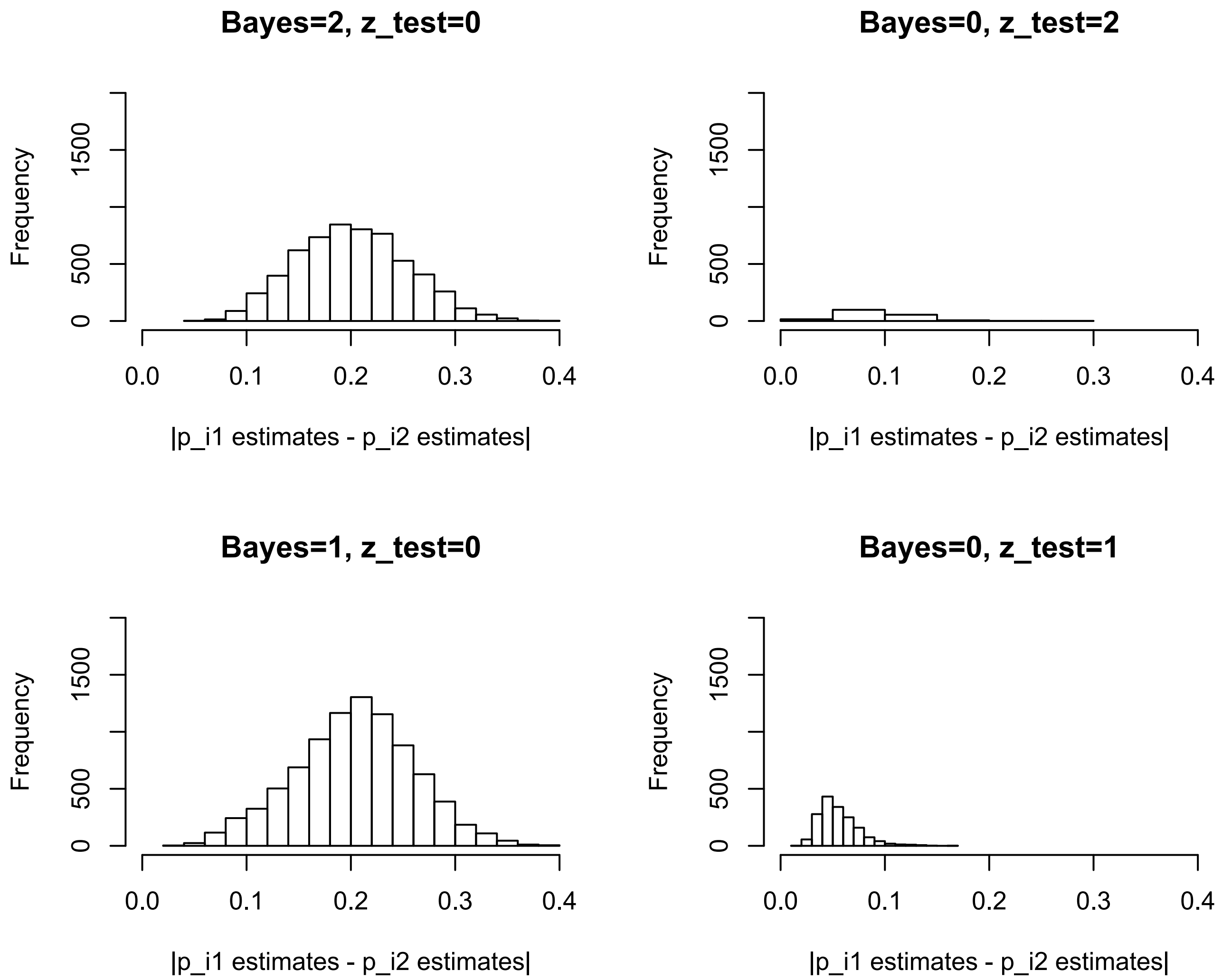

Figure S12 – Similar to Figure S11, except that FDR level for z test is controlled at 0.02.

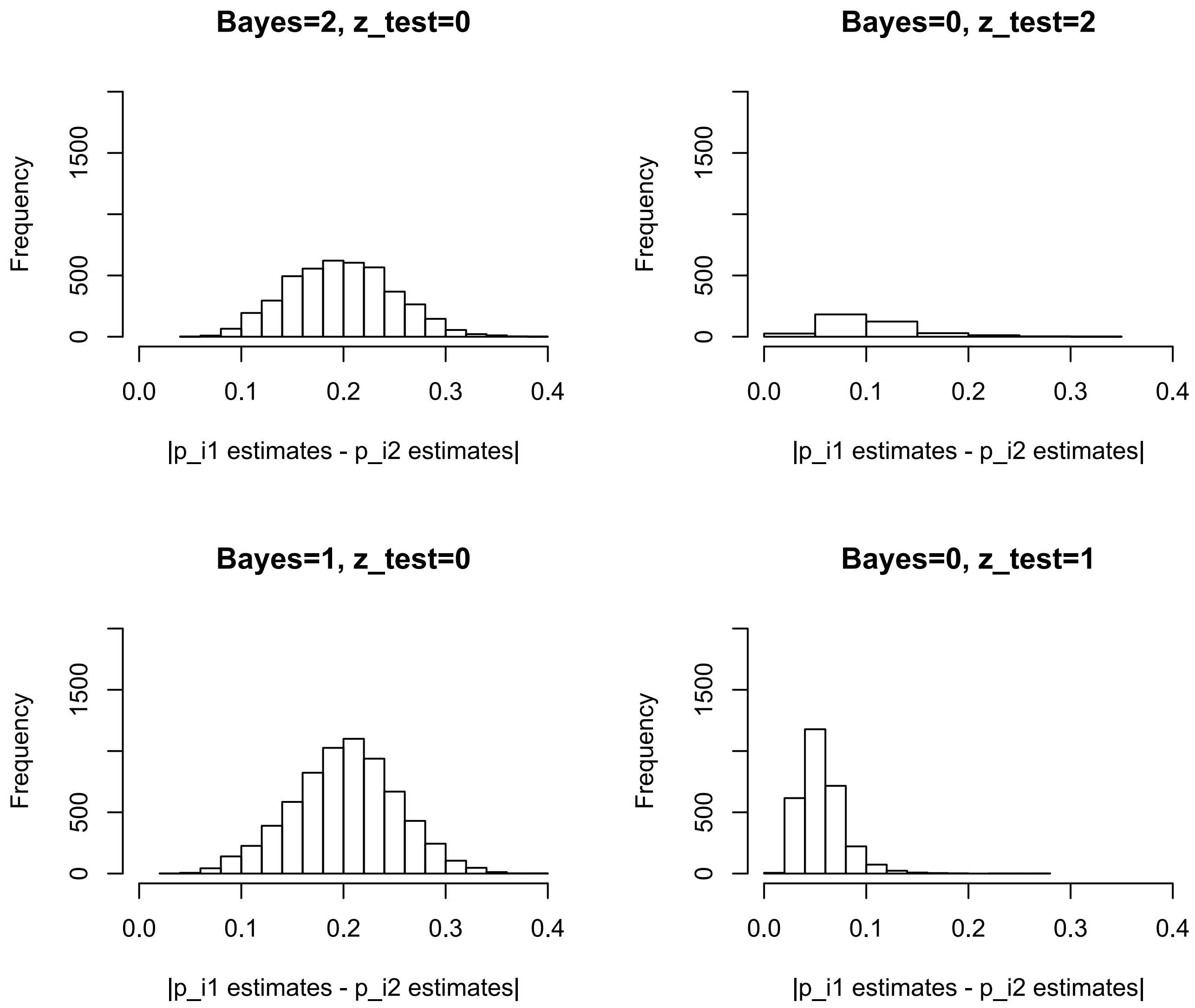

Figure S13 – Similar to Figure S11, except that FDR level for z test is controlled at 0.03.

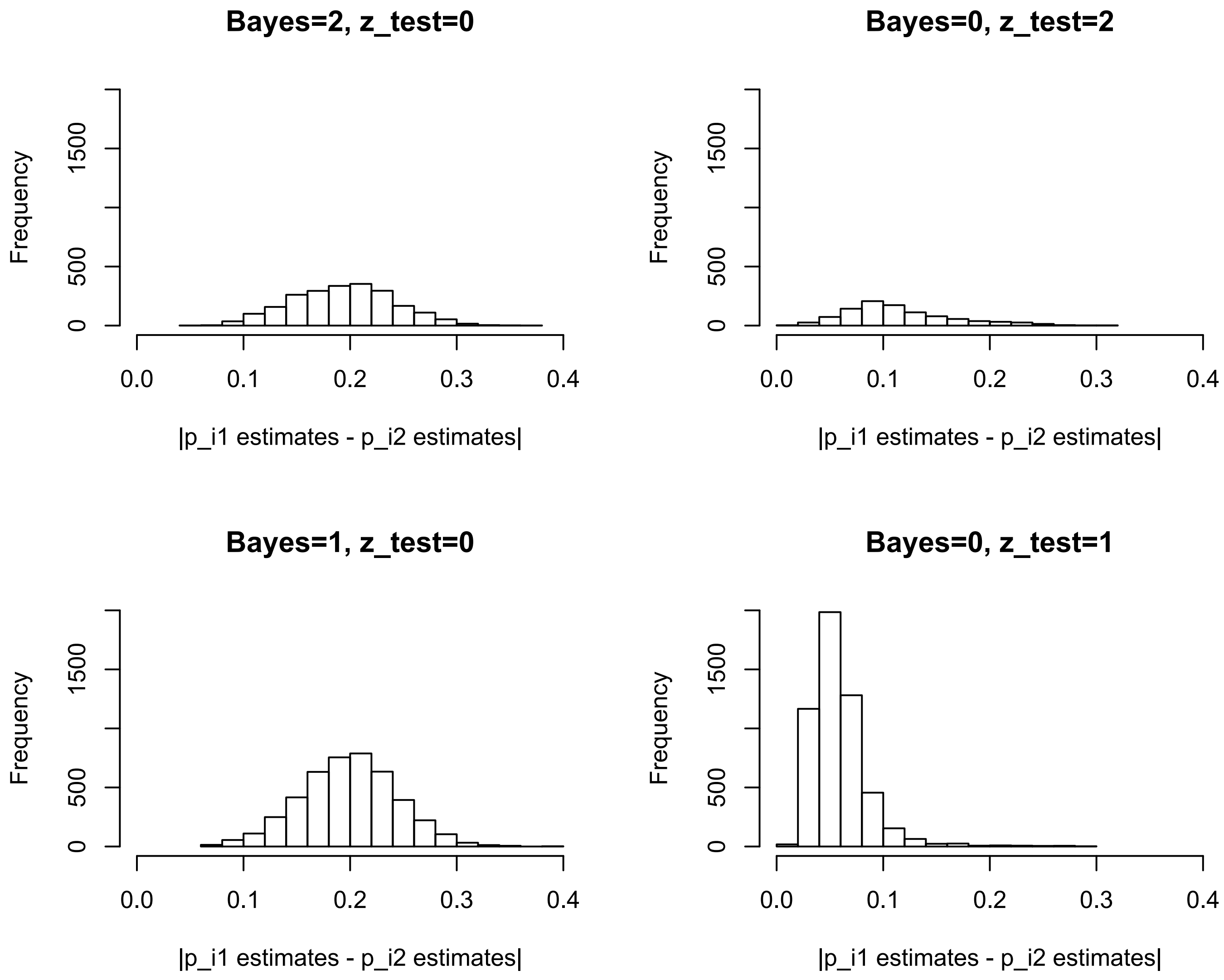

Figure S14 – Similar to Figure S11, except that FDR level for z test is controlled at 0.05.
